# Instantaneous sediment transport model for asymmetric oscillatory sheet flow

**DOI:** 10.1371/journal.pone.0190034

**Published:** 2017-12-22

**Authors:** Xin Chen, Yong Li, Genfa Chen, Fujun Wang, Liuchao Qiu

**Affiliations:** 1 Beijing Engineering Research Center of Safety and Energy Saving Technology for Water Supply Network System, China Agricultural University, Beijing, China; 2 Tianjin Centre, China Geological Survey, Tianjin, China; 3 Department of Water Resources Research, China Institute of Water Resources and Hydropower Research, Beijing, China; Tsinghua University, CHINA

## Abstract

On the basis of advanced concentration and velocity profiles above a mobile seabed, an instantaneous analytical model is derived for sediment transport in asymmetric oscillatory flow. The applied concentration profile is obtained from the classical exponential law based on mass conservation, and asymmetric velocity profile is developed following the turbulent boundary layer theory and the asymmetric wave theory. The proposed model includes two parts: the basic part that consists of erosion depth and free stream velocity, and can be simplified to the total Shields parameter power 3/2 in accordance with the classical empirical models, and the extra vital part that consists of phase-lead, boundary layer thickness and erosion depth. The effects of suspended sediment, phase-lag and asymmetric boundary layer development are considered particularly in the model. The observed instantaneous transport rate proportional to different velocity exponents due to phase-lag is unified and summarised by the proposed model. Both instantaneous and half period empirical formulas are compared with the developed model, using extensive data on a wide range of flow and sediment conditions. The synchronous variation in instantaneous transport rate with free stream velocity and its decrement caused by increased sediment size are predicted correctly. Net transport rates, especially offshore transport rates with large phase-lag under velocity skewed flows, which existing instantaneous type formulas failed to predict, are predicted correctly in both direction and magnitude by the proposed model. Net sediment transport rates are affected not only by suspended sediment and phase-lag, but also by the boundary layer difference between onshore and offshore.

## 1. Introduction

Nearshore asymmetric oscillatory flows are both velocity-skewed and acceleration-skewed as a result of wave propagation and transformation. When waves enter shallow waters, positive velocity skewness is produced; their wave crests sharpen, and their wave troughs flatten. When waves enter the surf zone, positive acceleration skewness is produced; the wave fronts steepen, and the wave rears become gentle. The sediment transport process becomes highly complex because the transport mechanisms are less straightforward under velocity-skewed and acceleration-skewed asymmetric oscillatory sheet flows. Accurate prediction of sediment transport is essential in environmental and morphological studies. Consequently, many studies have been conducted on sediment transport in asymmetric oscillatory sheet flows.

The studies are categorised into four types according to the wave shapes. The first is sinusoidal flow, which was studied by Refs [1–5]. The second type is pure velocity-skewed flow, which was studied by Refs [6–10]. The third type is pure acceleration-skewed flow, which was studied by Refs [2, 11, 12]. The fourth type is mixed flow with both velocity-skewness and acceleration-skewness, which was studied by Refs [13–16]. Both concentration and velocity profiles have been obtained and approximately expressed adequately [4, 17, 18]. Sediment size or suspension effect has also been discussed by [2, 5, 19, 20, 21]. The instantaneous sediment transport rate has been calibrated as a power function of velocity with different exponents [1, 3, 22]. Net sediment transport rate is extremely high as a consequence of high velocity skewness [20, 23, 24] or acceleration skewness [11–12]. All the aforementioned outstanding research achievements can provide a solid foundation for developing a general, unified and summarised analytical model.

Empirical models for sediment transport in oscillatory flows could be classified into quasi-steady models and semi-unsteady models [20, 25] according to phase-lag effects. In quasi-steady models such as those proposed by Refs [1, 26, 27, 28, 29], sediment transport is calculated immediately without considering the phase-lag between sediment movement and flow velocity. Semi-unsteady models such as the models established by Refs [6, 8, 11, 15, 25, 30, 31, 32, 33], consider the phase-residual or phase-shift. Acceleration effect (i.e., the contribution of phase-lag and asymmetric boundary layer development) also plays a vital role in the sediment transport and is considered in many quasi-steady or semi-unsteady models [11, 15, 25, 29, 31]. Theories about sediment movement have been established through these widely used models, but the prediction accuracy of such theories remains insufficient for a wide range of conditions, especially if more complex parameters are introduced. Most semi-unsteady models are half-period types without any information about the instantaneous sediment flux and transport rate. Furthermore, net current generated by the velocity skewness or acceleration skewness is still not straight considered. Despite of many instantaneous formulas, none of them can be applied for the analyzing of different power function exponents of velocity for instantaneous sediment transport rate because the phase-residual has not been included. Considering sediment flux distribution and sediment transport rate in instantaneous model is difficult, because many complex factors, such as suspended sediment, phase-lag and asymmetric boundary layer development, have to be reflected simultaneously. Currently, an instantaneous model that considers the above mentioned complex factors into both velocities and concentrations is still lacking.

Thus, this study attempts to derive the first instantaneous analytical model considering the effects of suspended sediment, phase-lag and boundary layer flow asymmetry particularly. The model is based on temporal and spatial approximation of both velocities and concentrations above a mobile bed, and can give near-bed sediment flux distribution and sediment transport rate in oscillatory flows. The different power function exponents of velocity for instantaneous sediment transport rate are expected to be uniformly expressed by the present model. The succeeding sections of this paper are organized as follows: velocity profile, concentration profile, erosion depth and the derivation for sediment transport are described in Section 2. Predictions and discussion about near-bed sediment flux and instantaneous and net sediment transport rates are presented in Section 3. Finally, the conclusions are drawn in Section 4.

## 2. Analytical model

In this study, the instantaneous sediment transport model is derived from the integration of concentration and velocity above the mobile seabed in relation to erosion depth.

### 2.1 Model derivation

**Fig 1:**
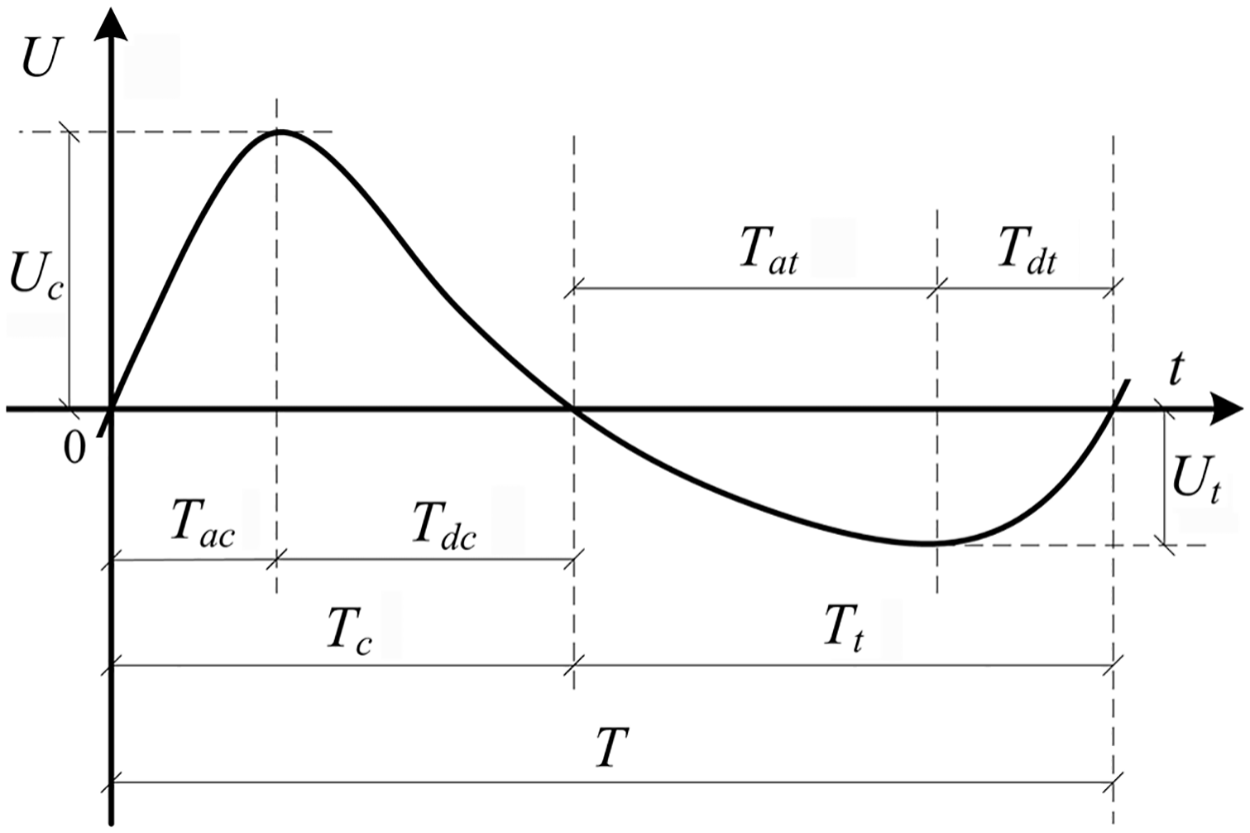
Free stream velocity duration.

In present study, the general form of Abreu [34] asymmetric free stream velocity (**W**) is applied as follows:

$$W(t)=\sum_{k=1}^{\infty }U_{k}\text{exp}[i(k\omega t+\varphi_{k})]=V+Ui$$ (1)

where the boldfaced notation represents a complex velocity; *t* is the time; *U*_*k*_ is the *k*^th^ harmonic velocity amplitude; *i* is the imaginary unit; *ω* = 2π/*T* is the angular frequency; *T* is the period; *φ*_*k*_ is the wave form parameter; *V* = Re(**W**); and *U* = Im(**W**). *U* is shown in Fig 1, where subscripts *c* and *t* are the crest and trough duration respectively; subscripts *a* and *d* respectively denote acceleration and deceleration duration; positive and negative symbols respectively denote onshore and offshore directions. The velocity in the wave boundary layer follows Nielsen and Guard [35], that is

$$W_{B}(y,t)=W\left\{1-\text{exp}\left[-(1+\alpha i)\frac{y+\Delta }{\delta }\right]\right\}$$ (2)

$$\text{Re}(W_{B})=\sum_{k=1}^{\infty }U_{k}\left[\text{cos}\left(k\omega t+\varphi_{k}\right)-\text{exp}\left(-\frac{y+\Delta }{\delta }\right)\text{cos}\left(k\omega t+\varphi_{k}-\alpha \frac{y+\Delta }{\delta }\right)\right]$$ (3)

$$\text{Im}(W_{B})=\sum_{k=1}^{\infty }U_{k}\left[\text{sin}\left(k\omega t+\varphi_{k}\right)-\text{exp}\left(-\frac{y+\Delta }{\delta }\right)\text{sin}\left(k\omega t+\varphi_{k}-\alpha \frac{y+\Delta }{\delta }\right)\right]$$ (4)

where subscript *B* denotes the boundary layer; *y* is the vertical coordinate; *y* = 0 is located at the initial undisturbed bed; *α* is the phase-lead parameter; Δ is the erosion depth; *δ* = *δ*_*B*_/4.6 is given by the turbulent boundary layer thickness *δ*_*B*_; and the amplitude of |**W**-**W**_***B***_|/|**W**| equals 0.01 at *y*+Δ = *δ*_*B*_. The velocity profiles [Eqs (2–4)] contain the erosion depth that denotes the immobile bed surface level. The phase-shift between *y* = 0 and *y* = -Δ is *α*Δ/*δ*. The present model is not valid for a ripple bed, a progressive wave or a wave–current condition because velocity profile Eqs (2–4) is only the approximation of oscillatory sheet flow.

For the model derivation, the real concentration distribution is ideally approached by the exponential law considering mass conservation [36], which is

$$S(y,t)=S_{m}\text{exp}\left[-(1+\frac{y}{\Delta })\right]$$ (5)

where *S* is the volumetric concentration; subscript *m* denotes the maximum value; and *S*_*m*_ = 0.6.

On the basis of velocity and concentration profiles in Eqs (2) and (5), the integration is

$$\begin{array}{l} \;\int_{-\Delta }^{\infty }S\left(y,t\right)W_{B}\left(y,t\right)dy\\ =\int_{-\Delta }^{\infty }S_{m}\text{exp}\left[-\left(1+\frac{y}{\Delta }\right)\right]W\left[1-\text{exp}\left(-\left(1+\alpha i\right)\frac{y+\Delta }{\delta }\right)\right]dy\\ =S_{m}W\int_{0}^{\infty }\text{exp}\left(-\frac{y\prime }{\Delta }\right)\left[1-\text{exp}\left(-\left(1+\alpha i\right)\frac{y\prime }{\delta }\right)\right]dy\prime \\ =S_{m}W\left[\Delta -{\frac{\delta \Delta }{\Delta \left(1+\alpha i\right)+\delta }\text{exp}\left(-y\prime \frac{\Delta \left(1+\alpha i\right)+\delta }{\delta \Delta }\right)|}_{\infty }^{0}\right]\\ =S_{m}\Delta W\frac{\left(1+\alpha i\right)}{\left(1+\alpha i\right)+\delta /\Delta } \end{array}$$ (6)

The wave boundary layer thickness *δ*_*B*_ = 4.6*δ* is directly obtained in Eq (6), where a large *δ*_*B*_ corresponds to a small shear stress and transport rate for the same flow [11]. The asymmetric *δ*_*B*_ generates net current and extra net sediment transport [37]. The following result is taken from the imaginary part of Eq (6), that is

$$\phi (t)=S_{m}\Delta \left\{\frac{U\left[(1+\alpha^{2})+\delta /\Delta \right]+V\alpha \delta /\Delta }{\alpha^{2}+{\left(1+\delta /\Delta \right)}^{2}}\right\}$$ (7)

*ϕ* is the instantaneous transport rate that related to the bottom velocity phase-lead, boundary layer thickness and erosion depth. The present harmonious summation of *U* and *V* in Eq (7) is caused by the phase-lead; however, the similar summation in Nielsen [29] is caused by acceleration and lead to an extra phase-shift. The dimensionless Eq (7) is

$$\Phi (t)=\frac{\phi }{\sqrt{(s-1)gD^{3}}}=S_{m}\frac{\Delta }{D}\frac{U\left[(1+\alpha^{2})+\delta /\Delta \right]+V\alpha \delta /\Delta }{\left[\alpha^{2}+{\left(1+\delta /\Delta \right)}^{2}\right]\sqrt{(s-1)gD}}$$ (8)

*S* = 0.08 is defined as the top of sheet flow layer [19], and located at *y* = Δ[-ln(0.08/*S*_*m*_)-1] *=* Δ according to Eq (5). The sediment transport rate in the sheet flow layer is

$$\begin{array}{ll} \int_{-\Delta }^{\Delta }SW_{B}dy & =S_{m}W{\left\{\Delta \text{exp}(-\frac{y}{\Delta })-\frac{\delta \Delta }{\Delta (1+\alpha i)+\delta }\text{exp}\left[-y\frac{\Delta (1+\alpha i)+\delta }{\delta \Delta }\right]\right\}|}_{2\Delta }^{0}\\ & =S_{m}\Delta W\left\{0.86-\frac{\delta }{\Delta (1+\alpha i)+\delta }\left[1-0.14\text{exp}(-\frac{2\Delta }{\delta })\text{exp}(-\frac{2\alpha \Delta i}{\delta })\right]\right\} \end{array}$$ (9)

Corresponding to free stream of *U*, the imaginary part is

$$S_{m}\Delta \left\{\begin{array}{l} U\left[0.86-\delta \frac{(\Delta +\delta )\left(1-0.14\text{exp}\left(-\frac{2\Delta }{\delta }\right)\text{cos}\left(\frac{2\Delta \alpha }{\delta }\right)\right)+0.14\alpha \Delta \text{exp}\left(-\frac{2\Delta }{\delta }\right)\text{sin}\left(\frac{2\Delta \alpha }{\delta }\right)}{{\left(\Delta +\delta \right)}^{2}+{\left(\alpha \Delta \right)}^{2}}\right]\\ +V\left[\delta \frac{\alpha \Delta \left(1-0.14\text{exp}\left(-\frac{2\Delta }{\delta }\right)\text{cos}\left(\frac{2\Delta \alpha }{\delta }\right)\right)-0.14(\Delta +\delta )\text{exp}\left(-\frac{2\Delta }{\delta }\right)\text{sin}\left(\frac{2\Delta \alpha }{\delta }\right)}{{\left(\Delta +\delta \right)}^{2}+{\left(\alpha \Delta \right)}^{2}}\right] \end{array}\right\}$$ (10)

### 2.2 Parameters

The boundary layer thickness, erosion depth and phase-lead are lacked in Eqs (1–10). To isolate the boundary layer asymmetry, *δ*_*B*_(*t*) is drawn from Fredsøe and Deiggard [38] and assumed to develop from flow reversal to flow peak [25, 31, 39] and from flow peak to reversal. Thus, *δ*_*B*_(*t*) is

$$\frac{\delta_{B}(t)}{k_{N}(t)}=0.09{\left[\frac{A(t)}{k_{N}(t)}\right]}^{0.82}$$ (11)

where *k*_*N*_(*t*)/*D =* 5Θ(*t*)≥1 is the roughness height over a mobile bed [40]; *D* is the sediment diameter. *A*(*t*) is the oscillatory flow orbital amplitude and is linearly interpolated between neighbouring flow peak (*A*_*c*,*t*_ = 2*U*_*c*,*t*_*T*_*ac*,*at*_*/π*) and flow reversal (*A*_*rc*,*rt*_ = 2*U*_*c*,*t*_*T*_*dc*,*dt*_*/π*, where *rc* and *rt* denote the flow reversal after flow crest and flow trough, respectively). The Shields parameter is defined as

$$\Theta (t)=\frac{fU^{2}}{2(s-1)gD}$$ (12)

where *s* is the sediment specific gravity; *g* is the gravitational acceleration; *f*(*t*) is the wave friction factor [40] modified by acceleration [15] and also linearly interpolated between neighbouring flow peak and flow reversal. The flow peak value (i.e., *f*_*c*_ and *f*_*t*_) are given by

$$6\pi \frac{T_{ac,at}}{T_{c,t}}\frac{A_{ac,at}}{k_{N}}=\sqrt{\frac{2}{f_{c,t}}}\text{exp}\left(0.4\sqrt{\frac{2}{f_{c,t}}}\right)\;\text{when}\;\frac{T_{ac,at}}{T_{c,t}}\frac{A_{ac,at}}{k_{N}}>0.39$$ (13)

The flow reversal value (i.e., *f*_*rc*_ and *f*_*rt*_) are given by replacing subscript *a* with *d* in Eq (13).

The erosion depth considering suspended sediment, phase-lag and asymmetric shear tress [41] is applied as follows

$$\frac{\Delta (t)}{D}=3.2\left[\alpha_{1}\Theta_{m}+\alpha_{2}\Theta (\omega t-\psi )\right]\left(0.011\frac{U_{m}}{w}+1\right)$$ (14)

where *α*_1_ = exp(-0.2/Ψ) is the phase-residual and *α*_1_+*α*_2_ = 1; *ψ* = Ψ is the phase-shift denoting Δ falling behind *U*; Ψ = *ωδ*_*S*_/*w* is the phase-lag parameter [19]; *δ*_*S*_ = 2Δ_*m*_ is the maximum sheet flow layer thickness; and *w* is the sediment falling velocity [42]. The phase-residual (*α*_1_) is approximated by the same exponential form as Eqs (2) and (5) by considering the two: (1) residual sediment amount is mildly converged to its maximum value and Δ(*t*) = Δ_*m*_ at the extremely large phase-lag (Ψ = ∞); (2) residual sediment amount is reduced to 0 and Δ(*t*)/Δ_*m*_ = Θ/Θ_*m*_ at the extremely small phase-lag (Ψ = 0). There should be a certain phase-shift between the maximum erosion depth and the maximum sheet flow layer thickness. Sediment above the initial bed is picked up from the area below the initial bed, and there is a concentration pivot near the initial bed [9]. The concentration variation above the pivot is in phase with *U* by following a phase-shift, whereas the variation below the pivot is in anti-phase with *U* by following a phase-shift. Notice the distance between the initial bed and the top of sheet flow layer is about Δ, and the distance between the initial bed and immobile bed surface is also Δ in the present application. Thus the phase-shift between the maximum erosion depth and the maximum sheet flow layer thickness is neglected.

Eq (8) reverts to the formula of Bailard [27] with Eq (14) if the phase-lead *α* is set to 0 and the variation of *δ/*Δ is neglected. If the suspended sediment is also discounted (Δ/*D*∝Θ), Eq (8) reverts to the classical Meyer-Peter and Müller [26] bedload formula Φ∝Θ^1.5^, which is also presented by Refs [28–29] that correspond to *ϕ*∝*fU*^3^ and Δ∝*fU*^2^. In the present study, the parameters’ sensitivity is not focused on. The actual phase-lead is about 15°–20° [3, 4, 10, 14]. So an averaged 18° is applied and *α* = tan18° = 0.32.

By now the instantaneous transport rate Eqs (6–10) have been confirmed since all the parameters (*δ*_*B*_, Δ and *α*) are given. *ϕ*(*t*) is usually expressed as *ϕ/ϕ*_*m*_ = Sign(*U*)|*U/U*_*m*_|^*n*^. Various exponents *n* = 1–4 were obtained [1, 3, 22] and summarised with *α* = 0, i.e., *n* increases as the decrement of phase-lag. In this case, Eq (7) reverts to:

$$\;\phi (t)=\frac{S_{m}\Delta U}{1+\delta /\Delta }$$ (15)

If the phase-lag effect is extremely large (*α*_1_ = 1), then Δ(*t*) = Δ_*m*_ and Eq (15) becomes

$$\phi (t)=\frac{S_{m}U\Delta_{m}}{1+\delta /\Delta_{m}}$$ (16)

Thus, *ϕ/ϕ*_*m*_ = *U*/*U*_*m*_ corresponds to *n* = 1. This result agrees with the large phase-lag case T5R1 with *α*_1_ = 0.92 [3]. If the phase-lag is minimal (*α*_1_ = 0), then Eq (14) becomes Δ = *α*_Δ_*U*^2^, where *α*_Δ_ = Δ_*m*_*/U*_*m*_^2^. Eq (15) becomes

$$\phi (t)=\frac{S_{m}\alpha_{\Delta }{}^{2}U^{5}}{\delta +\alpha_{\Delta }U^{2}}$$ (17)

**Fig 2:**
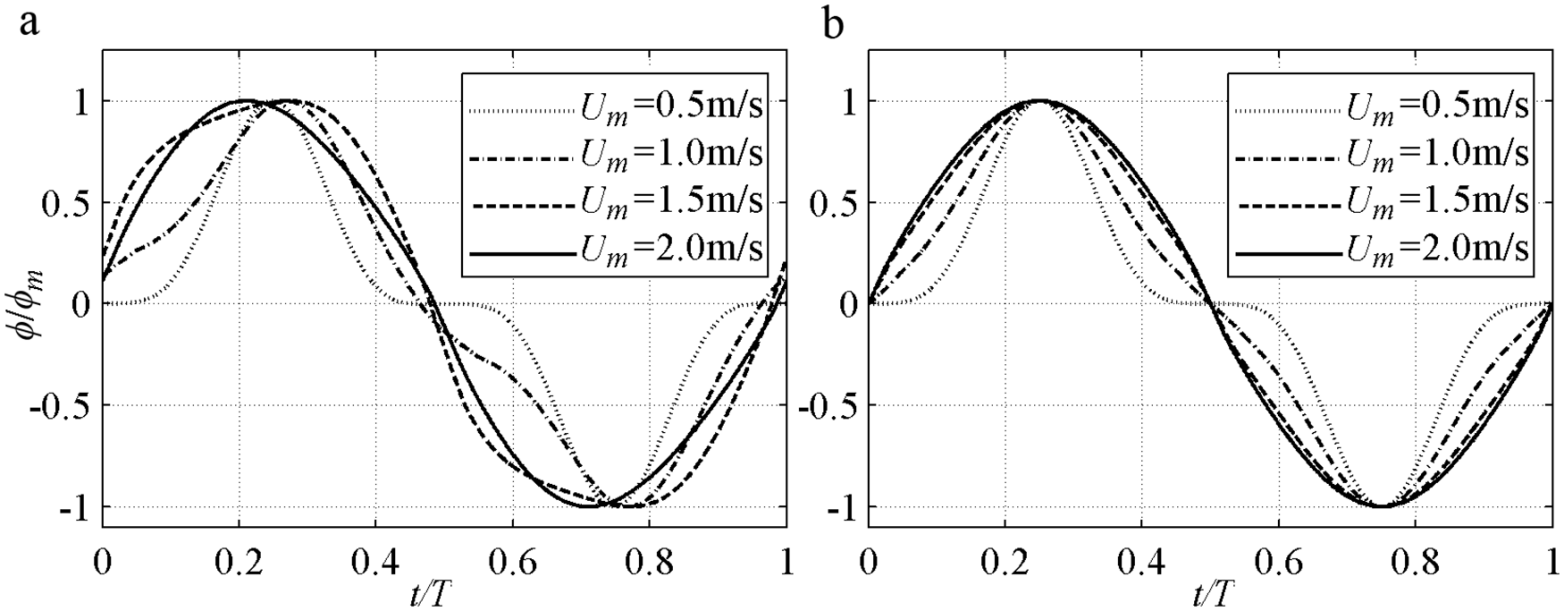
Instantaneous sediment transport rate of sinusoidal flow predicted by Eq (7). a) With phase-lead and phase-shift; b) Without phase-lead and phase-shift.

At the flow reversal, *ϕ/ϕ*_*m*_ = (*U*/*U*_*m*_)^*5*^ and *n* = 5. At the other time, *n* = 3–5 because *U*^2^ exists in the denominator. This result is almost in agreement with *n* = 4 found in the minimal phase-lag experiment [1] with large *D*, small *U*_*m*_ and *α*_1_<0.1. Fig 2a shows a series simulation of *ϕ*(*t*)*/ϕ*_*m*_ in Eq (7) based on the sinusoidal experiment of O’Donoghue and Wright [9]. In the said experiment, *D* = 0.13mm and *T* = 5s with an increment of phase-lag caused by *U*_*m*_ = [0.5, 1.0, 1.5, 2.0]m/s. The 1.0m/s and 1.5m/s in Fig 2a show different features in a half cycle because the phase-lead causes slightly large *ϕ* after flow reversal, and the phase-shift enlarges the local *ϕ* near the time corresponding to maximum erosion depth. For improved clarity, Eq (7) without phase-shift and phase-lead is shown in Fig 2b. *ϕ*(*t*)*/ϕ*_*m*_ at the crest becomes plump and flattens when *n* decreases from 4 to 1, when the phase-lag and *α*_1_ = [0.04, 0.53, 0.82, 0.92] are increased by *U*_*m*_. Generally, the curves can be approximated by Sign(*U*)|*U/U*_*m*_|^*n*^ with *n* = 1 for *α*_1_>0.8, *n* = 2 for *α*_1_ = 0.4–0.5 and *n* = 3–5 for *α*_1_<0.2. *n* for Eq (7) is slightly larger than that in [37] and can extend to 3–5 due to the *δ* that can present the net current and extra net sediment transport in asymmetric flow.

## 3. Results

**Fig 3:**
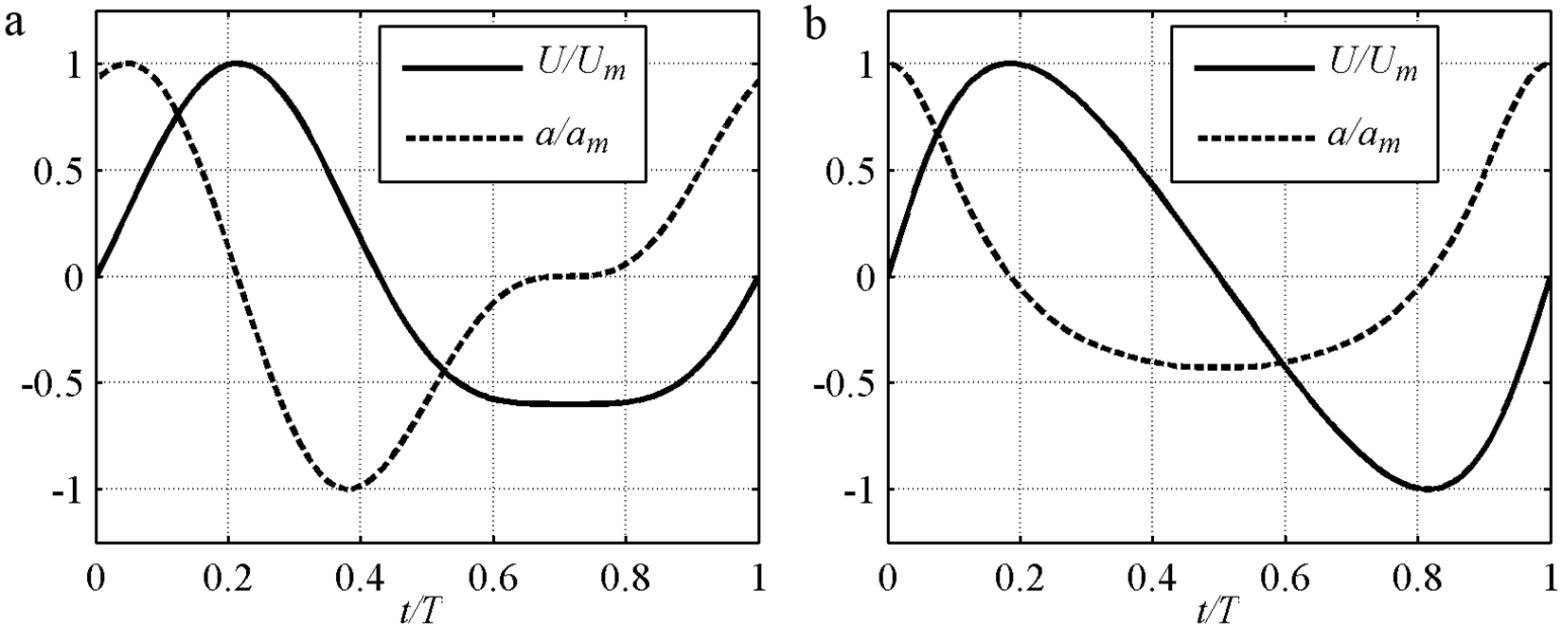
Free stream velocity of asymmetric oscillatory flow. a) Pure velocity-skewed flow; b) Pure acceleration-skewed flow.

The present model is applied for different wave shapes (Fig 3) to isolate the effects of skewed velocity and skewed acceleration. Experiments selected for comparison [Table 1][2, 6, 7, 8, 9, 10, 11, 12, 14, 15, 24, 43] include the pure velocity-skewed flow in Section 3.1, the pure acceleration-skewed flow in Section 3.2, and the mixed velocity- and acceleration-skewed flow in Section 3.3. Net *ϕ* results for different wave shapes are in Section 3.4. In pure velocity-skewed flow, <*U*^3^>≠0, <*a*^3^> = 0 and acceleration degree *β* = *a*_max_/(*a*_max_+*a*_min_) = 0.5, where *a* = *dU*/*dt* and the angle brackets denote the periodic average. In pure acceleration-skewed flow, <*a*^3^>≠0, <*U*^3^> = 0, and velocity asymmetry *R* = *U*_*c*_/(*U*_*c*_+*U*_*t*_) = 0.5. Several classical formulas are also used for comparison. The instantaneous formulas are Ribberink [28], which did not consider boundary layer asymmetry, and Nielsen [29], which accounted for shear stress asymmetry. The half-period formulas are Watanabe and Sato [11] and Silva et al. [25] accounting for shear stress asymmetry and phase-lag. In the results, data is abbreviated as ‘Exp.’; the present model is abbreviated as ‘Pres.’; the other formulas are abbreviated as ‘R98’, ‘S06’, ‘W04’ and ‘N06’.

**Table 1:** Data for sediment transport rate validation.

Authors	Flow type	*D*(mm)	*U*_*m*_(m/s)	*T*(s)	Number
Ahmed and Sato [8]	Cnoidal	0.21–0.74	1.16–1.85	3.0	15
Dibajnia [6]	Cnoidal	0.20	0.71–1.39	1.0–4.0	25
Dong et al. [15]	Mixed	0.16~0.3	0.77–1.68	3.0–7.0	35
King [2]	Sinusoidal	0.135–1.1	0.30–1.22	2.0–12.0	74
Li et al. [43]	Stokes	0.13	1.25–1.5	4.0–6.0	2
O’Donoghue and Wright [9–10]	Stokes	0.13–0.46	1.50	5.0–7.5	6
Ribberink and Al-Salem [7, 24]	Stokes	0.21	0.60–1.70	5.0–12.0	16
Ruessink et al. [14]	Mixed	0.20	1.2–1.44	7.0	3
vander A et al. [12]	Sawtooth	0.15–0.46	0.83–1.30	5.0–9.0	35
Watanabe and Sato [11]	Sawtooth	0.2–0.74	0.84–1.45	3.0–5.0	33

### 3.1 Pure velocity-skewed flow

**Fig 4:**
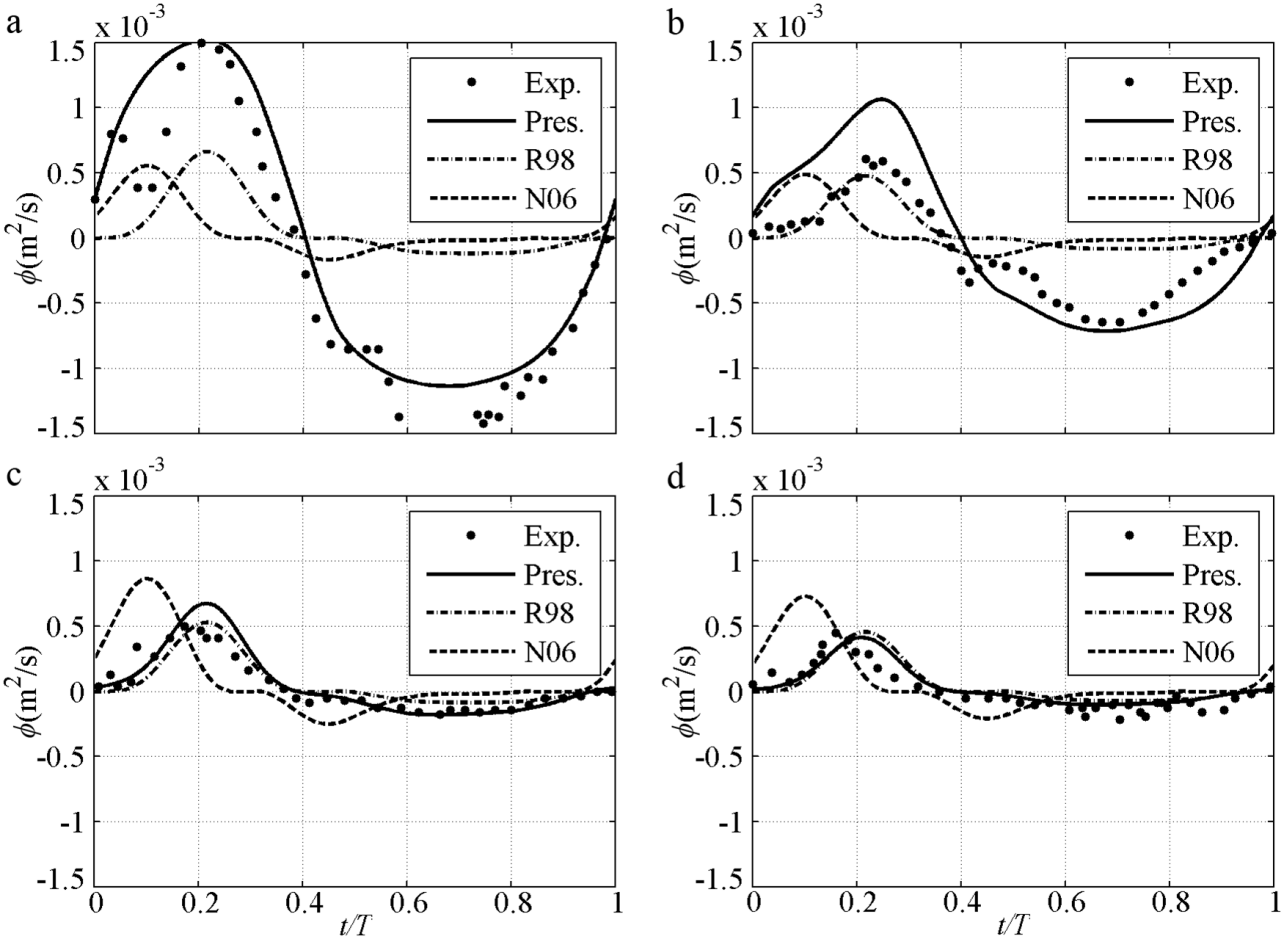
Instantaneous transport rate for pure velocity-skewed flow with *U*_*m*_ = 1.5m/s and *R* = 0.625. a) FA5010: *D* = 0.13mm, *T* = 5.0s; b) FA7515: *D* = 0.13mm, *T* = 7.5s; c) CA5010: *D* = 0.46mm, *T* = 5.0s; d) CA7515: *D* = 0.46mm, *T* = 7.5s.

An instantaneous process can clearly illustrate the advantages of the proposed model in determining suspended sediment, phase-lag and boundary layer asymmetry, as well as the contributions of these factors to the net sediment transport rate. The instantaneous sediment transport rate under 2^nd^ Stokes flow is first shown (Fig 4), in which *U*_*m*_ = *U*_*c*_ = 1.5m/s, *U*_*t*_ = 0.9m/s, *D* = 0.13–0.46mm, *T* = 5.0–7.5s and *R* = 0.625 [9–10]. The predictions by the proposed model are satisfactory in all cases in which the *ϕ* variation almost follows *U* in Fig 3a. Firstly, owing to decreased Δ with suspended sediment caused by increased *D* in Eq (14), only the proposed model can predict the decreasing tendency of the transport rate from the fine case to the coarse case. Secondly, in the proposed model, residual Δ caused by phase-lag is included [Eq (14)] as *α*_1_Θ_*m*_. Thirdly, a relatively large offshore *U*_*B*_ is caused by a relatively small wave boundary layer thickness (*δ*_*B*_) as a result of the relatively small *k*_*N*_ in Eq (11).

**Fig 5:**
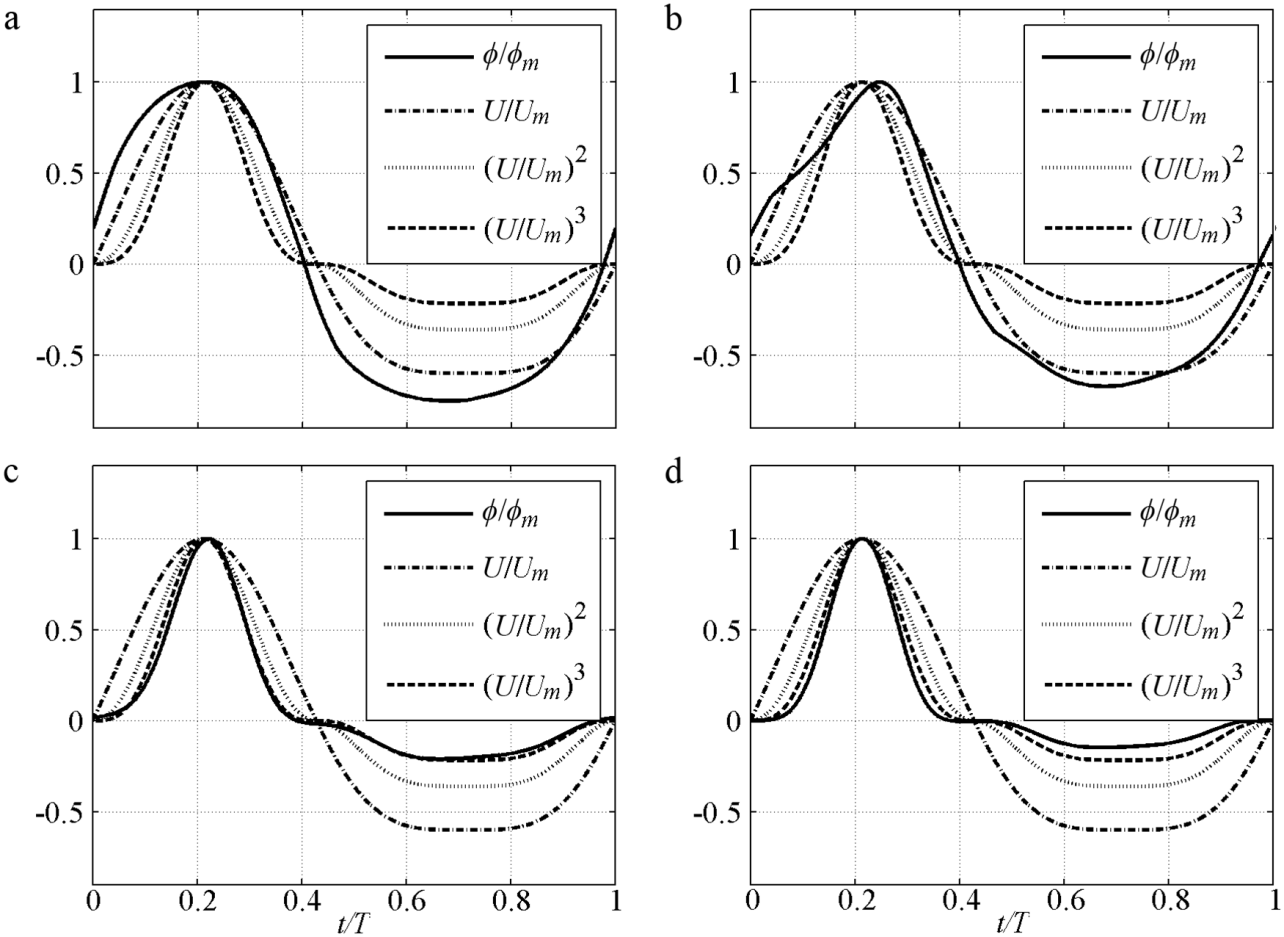
Comparison instantaneous transport rate with *U* for pure velocity-skewed flow with *U*_*m*_ = 1.5m/s and *R* = 0.625, (*U*/*U*_*m*_)^*2*^ at offshore stage is mirrored opposite for comparison. a) FA5010: *D* = 0.13mm, *T* = 5.0s; b) FA7515: *D* = 0.13mm, *T* = 7.5s; c) CA5010: *D* = 0.46mm, *T* = 5.0s; d) CA7515: *D* = 0.46mm, *T* = 7.5s.

The phase-lag effect of fine FA5010 (*α*_1_ = 0.83) and FA7515 (*α*_1_ = 0.71) is highly significant. Consequently, Δ in the offshore duration is close to that in the onshore duration. Even *U*_*t*_ is much smaller than *U*_*c*_, the *ϕ* near the flow trough (*t/T* = 0.6–0.8) is also close to that near the flow crest (*t/T* = 0.15–0.25). The phase-lag effect is important for offshore net sediment transport rate, as observed by [10]. In addition, the boundary layer thickness (*δ*_*B*_) in the offshore duration should be smaller than that in the onshore duration because the offshore Θ and *k*_*N*_ are much smaller than that at onshore [40], and *δ*_*B*_ is proportional to *k*_*N*_ [38, 42], thereby leading to a relatively large *U*_*B*_ in the offshore duration in Eq (4). Thus, the periodic averaged *U*_*B*_ (net current) is offshore in O’Donoghue and Wright [10], and the offshore net *ϕ* is generated. This process is clearly illustrated [Fig 5a and 5b] with a comparison to (*U*/*U*_*m*_)^*n*^. An extremely large phase-lag and a constant *δ*_*B*_ allow *ϕ/ϕ*_*m*_ = *U*/*U*_*m*_ to be used in these two cases, which are close to those in Dick and Sleath [3] of $\int_{0}^{T}\phi /\phi_{m}dt\approx \int_{0}^{T}U/U_{m}dt=0$ that offshore net *ϕ* will not appear. But the offshore *U*_*B*_ in the pure velocity-skewed flow is relatively large because of the small *δ*_*B*_, which results in *ϕ/ϕ*_*m*_<*U*/*U*_*m*_ near the flow trough (*t/T* = 0.6–0.8) [Fig 5a and 5b]. So the net *ϕ* is offshore: $\int_{0}^{T}\phi /\phi_{m}dt<\int_{0}^{T}U/U_{m}dt=0$. The effect of velocity skewness is the contribution of phase-lag and boundary layer asymmetry. The predictions obtained by using the formulas [28–29] are inadequate [Fig 4a and 4b] without suspension sediment, phase-lag and boundary layer thickness. Here, the phase-shift presented by Nielsen [29] is caused by the summation of *U* and *dU/dt* (its representative free stream velocity).

In the coarse cases, the formulas [28–29] can be adequately used because the suspension amount and phase-lag are much smaller than those of the fine sediment case. For the coarse CA5010 (*α*_1_ = 0.18) and CA7515 (*α*_1_ = 0.05) with small phase-lag, sediments are difficult to pick up as the flow velocity increases, and they descend easily as the flow velocity decreases. The *ϕ/ϕ*_*m*_≈(*U*/*U*_*m*_)^3^, which is close to the formulas [28–29] without phase-lag in theory, can be used to approximate the present result [Fig 5c and 5d]. The Δ near the flow peak (*t/T* = 0.15–0.25) is significantly greater than that near the flow trough (*t/T* = 0.6–0.8) according to Eq (14). Thus, a much stronger *ϕ*_*c*_ than *ϕ*_*t*_ in the sheet flow layer is generated [Fig 4c and 4d], and leads to an onshore net sediment transport rate of $\int_{0}^{T}\phi /\phi_{m}dt\approx \int_{0}^{T}{\left(U/U_{m}\right)}^{3}dt>0$.

**Fig 6:**
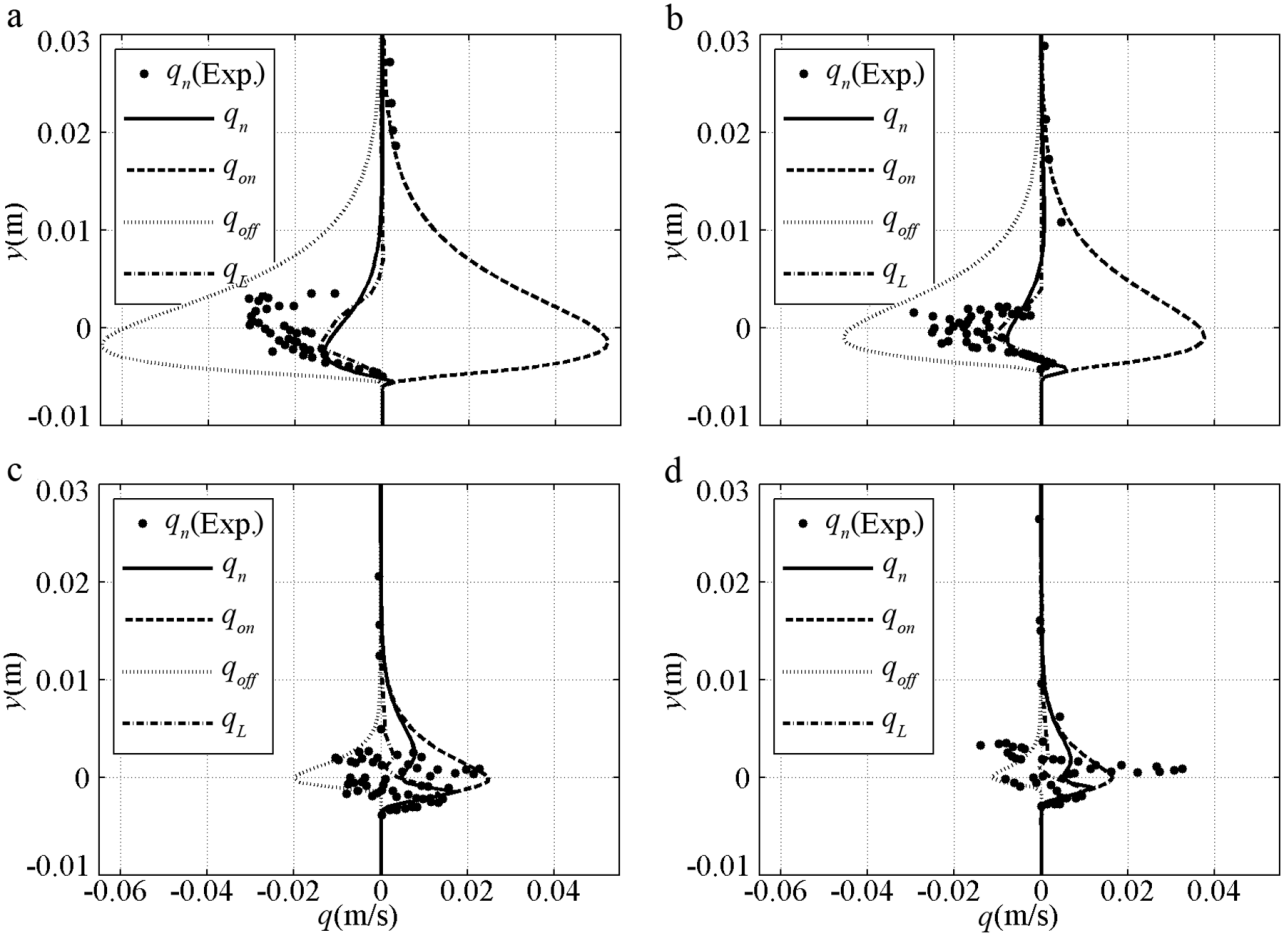
Sediment flux validation for pure velocity-skewed flow with *U*_*m*_ = 1.5m/s and *R* = 0.625. a) FA5010: *D* = 0.13mm, *T* = 5.0s; b) FA7515: *D* = 0.13mm, *T* = 7.5s; c) CA5010: *D* = 0.46mm, *T* = 5.0s; d) CA7515: *D* = 0.46mm, *T* = 7.5s.

A time-averaged sediment flux is shown in Fig 6 to validate the present model at different elevations. In Fig 6, $q_{on}(y)=\int_{0}^{Tc}S(y,t)U_{B}(y,t)dt/T$ and $q_{off}(y)=\int_{Tc}^{T}S(y,t)U_{B}(y,t)dt/T$ are the total onshore flux and offshore flux respectively. *q*_*n*_ = *q*_*on*_+*q*_*off*_ is the total averaged sediment flux, which corresponds to *q*_*L*_ from the two-phase model of Liu and Sato [39]; the black dots denote the experiment. The bottom level of the flux profile denotes the location of the maximum erosion depth, Δ_*m*_. For the fine [Fig 6a and 6b] cases, the periodic variations in Δ and concentration are minimal as a result of the large phase-lag; thus, the bottom levels of *q*_*on*_ and *q*_*off*_ are almost the same. As a result of relatively small *U*_*B*_ in onshore duration corresponding to large *δ*_*B*_, *q*_*on*_ is less than *q*_*off*_ at each elevation, and *q*_*n*_ is negative. The prediction reproduces the features with underestimated *q*_*n*_ and agrees with the offshore underestimation of FA5010 and onshore overestimation of FA7515 in Fig 4. However, the prediction is considered acceptable compared with the underestimations by the two-phase model of Liu and Sato [39]. For the coarse sediment cases, onshore Δ is much larger than offshore Δ, and the bottom level of *q*_*on*_ is much lower than that of *q*_*off*_. When more sediment is transported in onshore duration, *q*_*on*_ is larger than *q*_*off*_, and *q*_*n*_ is positive. Above the initial bed, the data are scattered because of concentration measurement uncertainty. However, the *q*_*n*_ predicted by the present model almost passes the centre of the data, and the flux profiles are similar to those of Liu and Sato [39].

**Fig 7:**
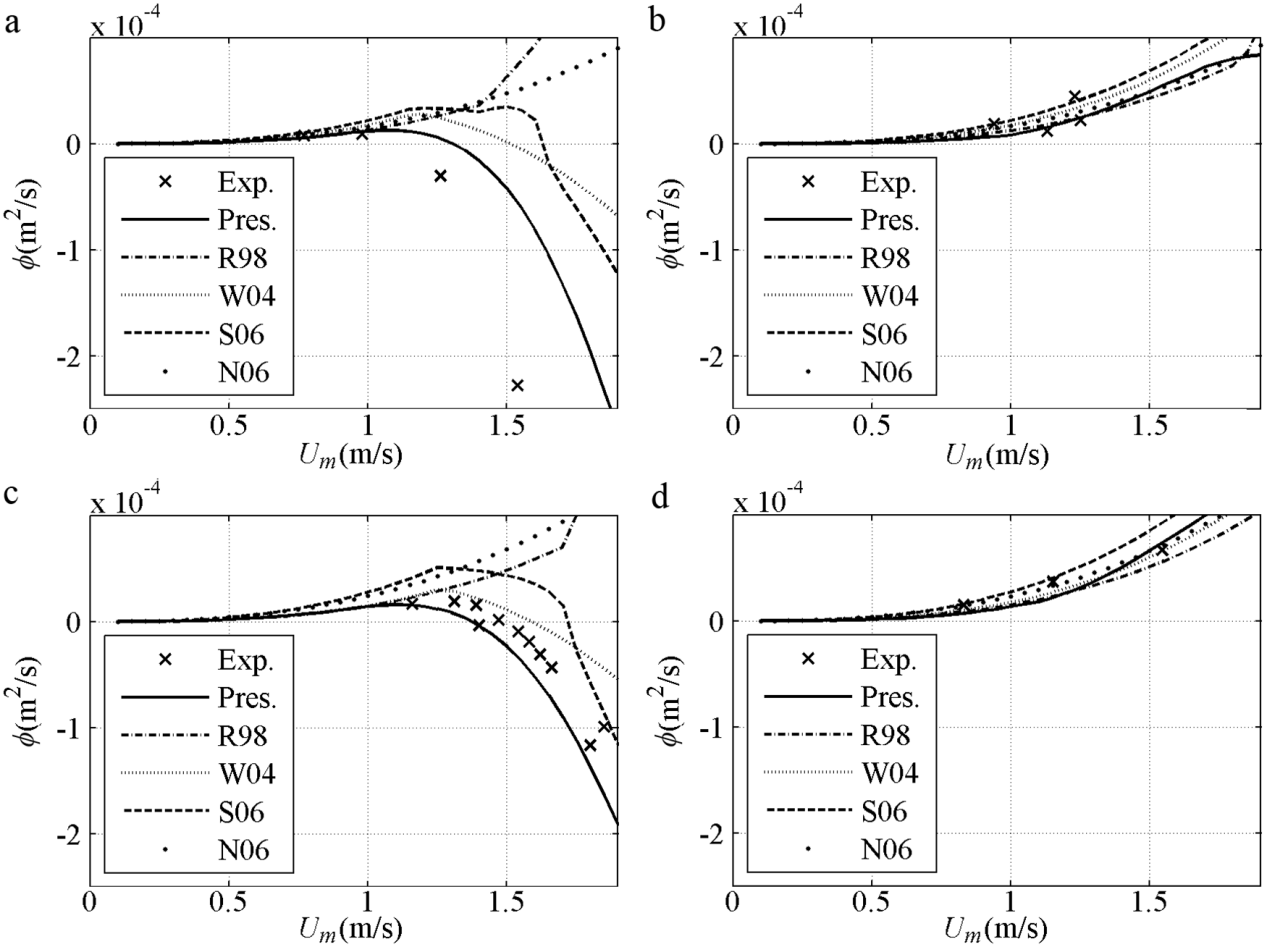
Validation of net sediment transport rates against *U*_*m*_ for pure velocity-skewed flow. a) *D* = 0.13mm, *T* = 6.5s, *R* = 0.62; b) *D* = 0.21mm, *T* = 9.5s, *R* = 0.62; d) *D* = 0.21mm, *T* = 3s, *R* = 0.60; d) *D* = 0.32mm, *T* = 6.5s, *R* = 0.62.

After validation based on the same *U*_*m*_, the net sediment transport rates against *U*_*m*_ are shown, including the 2^nd^ Stokes flows [44] in Fig 7a and 7b and [45] in Fig 7d and the 1^st^ Cnoidal flows [8] in Fig 7c. The predicted net *ϕ* is onshore and in agreement with the experimental result when *U*_*m*_ (phase-lag) is small in each formula. The net *ϕ* decreases to offshore when *U*_*m*_ (phase-lag) is considerable with fine *D* = 0.13mm [Fig 7a], or short *T* = 3s even if *D* = 0.21mm>0.20mm [Fig 7c] that is thought positive in van der A et al. [32]. Such tendency can be predicted by the present model with the use of phase-lag and asymmetric boundary layer. The insufficient offshore quantity obtained by Watanabe and Sato [11] and Silva et al. [25] is probably due to their failure in the averaged offshore *U*_*B*_ (net current). The appearance of the phase-lag in the present model is continuous. By contrast, the phase-lag is suddenly triggered in Watanabe and Sato [11] and Silva et al. [25] when Ω_c_*>0, leading to a constant sediment amount carried in the *T*_*c*_ duration until a sufficiently large *U*_*t*_ triggers Ω_t_*>0. Ω_c_* (Ω_t_*) denotes the sediment carried up by present half cycle *c* (*t*) but taken away by next half cycle. Turning points for the formulas exist when *U*_*m*_ is considerable: (1) in Ribberink [28], *k*_*N*_/*D*>1 is achieved; (2) in Watanabe and Sato [11], Ω_c_*>0 is obtained and phase-lag effect appears; (3) in Silva et al. [25], Ω_c_*>0 or *k*_*N*_/*D*>1 is attained. In Fig 7b and 7d, the net *ϕ* in each formula is onshore and has an increasing tendency before *U*_*m*_ is 1.5m/s because of small phase-lag with long *T* = 9.5s or coarse *D* = 0.32mm. Overall, the formulas are in good agreement with the experiments during the onshore increment stage with small phase-lag, corresponding to an instantaneous approach, i.e. *ϕ/ϕ*_*m*_≈(*U*/*U*_*m*_)^3^. As mentioned by Al-Salem [8], the formulas almost obey the same <*ϕ*>∝<*U*^3^> at this stage.

**Fig 8:**
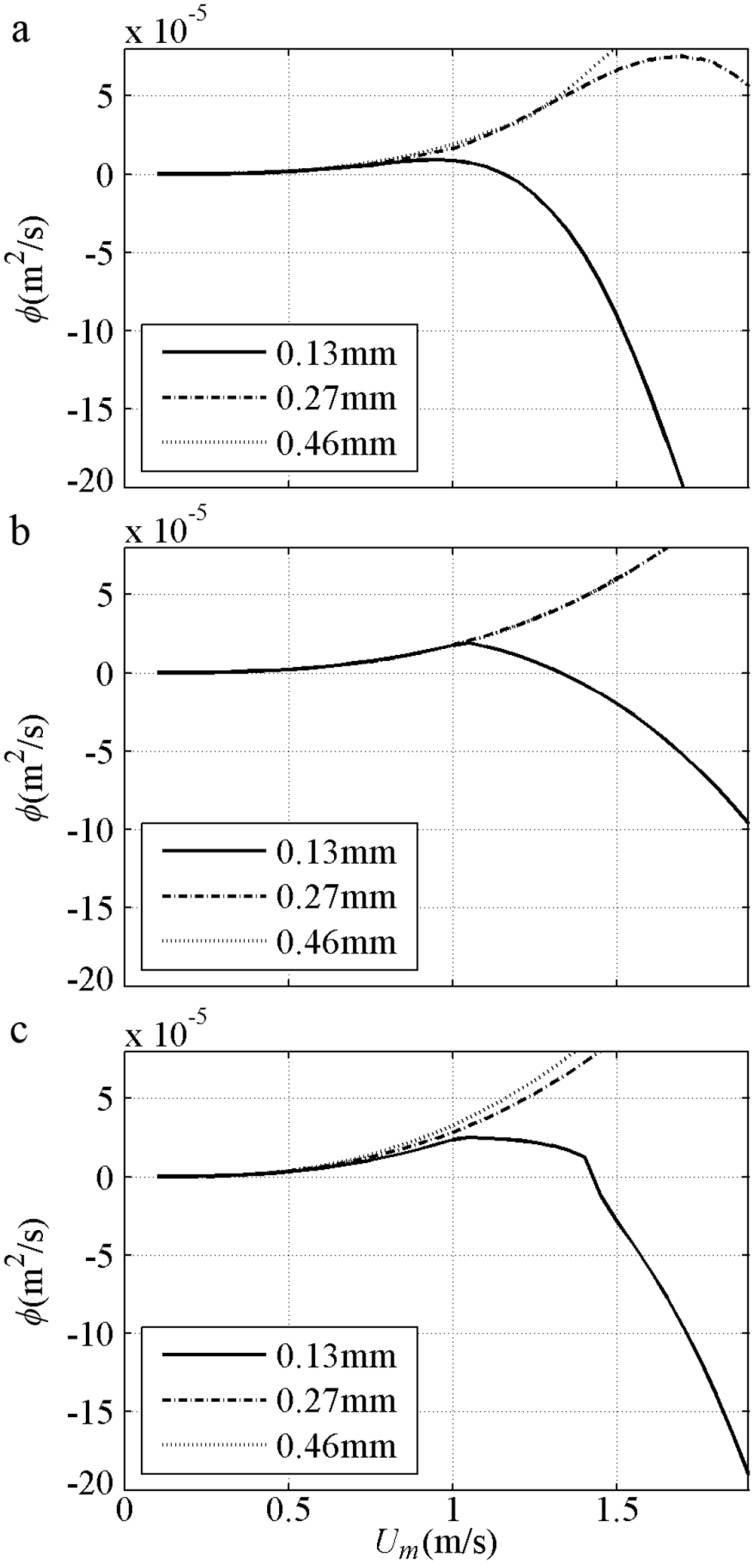
Net sediment transport of 2^nd^ Stokes flows grouped by *D* with *T* = 5s and *R* = 0.625. a) Present model; b) Watanabe and Sato (2004); c) Silva et al. (2006).

Sediment size effect about net *ϕ* are shown in Fig 8 with *T* = 5s and *R* = 0.625 for 2^nd^ Stokes flows [9–10]. For the fine (0.13mm) case, the net *ϕ* variation is similar to that in Ribberink and Chen [44] [Fig 7a]. For the medium (0.27mm) case, the onshore net *ϕ* initially increases to a specific maximum and then assumes a realistic decreasing tendency, because a proper phase-lag exists when a minimum Δ = 4*D* at *U*_*m*_ = 1.5m/s is measured by O’Donoghue and Wright [9]. The offshore net *ϕ* in the medium sediment can also be observed when the phase-lag is sufficiently large, as in Ahmed and Sato’s [8] experiments [Fig 7c]. For the coarse (0.46mm) case, the net *ϕ* variation is similar to that in Hassan and Ribberink [45] [Fig 7d]. The net *ϕ* does not simply increase or decrease with *D* under the same *U*_*m*_ for pure velocity-skewed flow. At the same small *U*_*m*_, the net *ϕ* of all *D* values are close to each other because the suspension amount and phase-lag are small. At the same large *U*_*m*_ with phase-lag, the net *ϕ* for the fine case are the smallest and offshore. The RANS results of Hassan and Ribberink [20] with *R* = 0.62, *T* = 6.5s and *U*_*m*_<1.6m/s show that the tendency of net *ϕ* against *D* in Fig 8a is realistic probably because of the continuous appearance of the phase-lag without triggering Ω_c_*>0 in Fig 8b and 8c.

### 3.2 Pure acceleration-skewed flow

**Fig 9:**
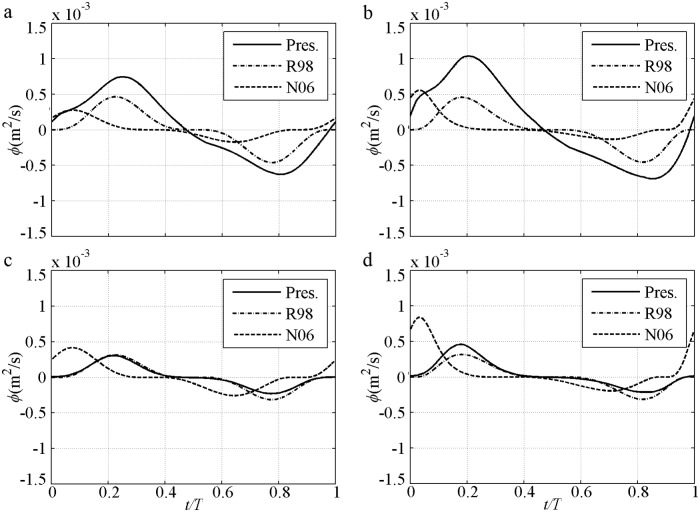
Instantaneous sediment transport rate for pure acceleration-skewed flow with *U*_*m*_ = 1.3m/s. a) S556015f: *D* = 0.15mm, *β* = 0.58; b) S706015f: *D* = 0.15mm, *β* = 0.70; c) S556015c: *D* = 0.46mm, *β* = 0.58; d) S706015c: *D* = 0.46mm, *β* = 0.71.

**Fig 10:**
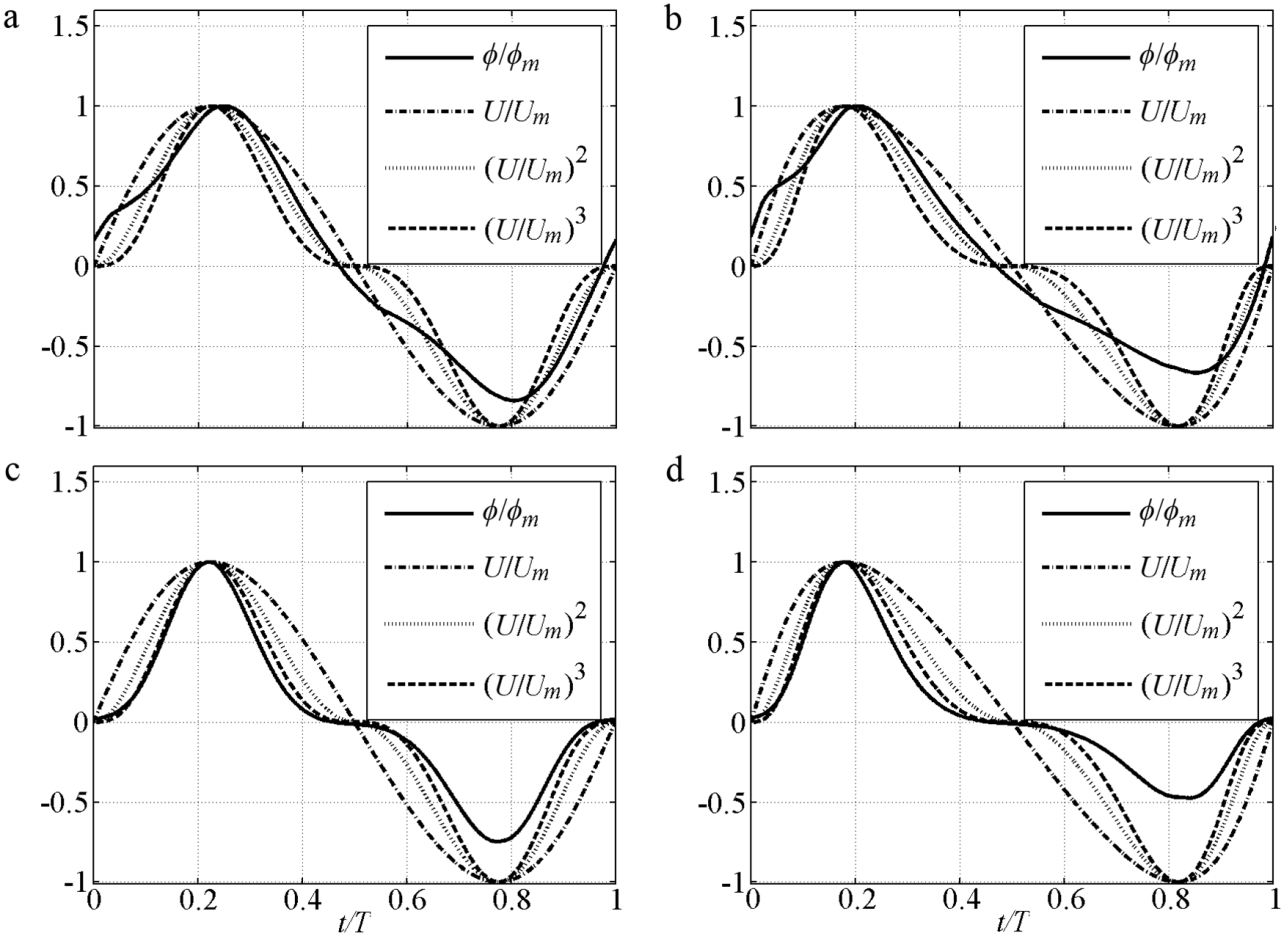
Comparison instantaneous transport rate with *U* for pure acceleration-skewed flow with *U*_*m*_ = 1.3m/s, (*U*/*U*_*m*_)^2^ at offshore stage is mirrored opposite for comparison. a) S556015f: *D* = 0.15mm, *β* = 0.58; b) S706015f: *D* = 0.15mm, *β* = 0.70; c) S556015c: *D* = 0.46mm, *β* = 0.58; d) S706015c: *D* = 0.46mm, *β* = 0.71.

Sawtooth flows of *U*_*m*_ = *U*_*c*_ = *U*_*t*_ = 1.3m/s, *T* = 6s, *D* = 0.15–0.46mm and *β* = 0.58–0.71 [12] are initially selected for the study. The instantaneous *ϕ* is shown in Fig 9, and a comparison of (*U*/*U*_*m*_)^*n*^ with the present model is shown in Fig 10. Predicted instantaneous *ϕ* by the present model follows *U* in Fig 3b well, and the *ϕ*_*c*_ is always larger than the *ϕ*_*t*_. At the same *D* in the present model, a larger *β* corresponds to a larger *ϕ*_*c*_ [Fig 9], and a smaller *ϕ*_*t*_ [Fig 10], and thus a larger positive net *ϕ*.

When *D* increases, the present predicted *ϕ* decreases in agreement with Fig 4 because Δ in Eq (14) decreases with a decreased suspension amount. Instantaneous experimental data are unavailable; however the validation of the net *ϕ* can be seen in the later discussion. The onshore net *ϕ* and its increment with decreasing *D* and increasing *β* are observed by vander A et al. [12]. As illustrated by vander A et al. [12, 46], the boundary layer has less time to develop before *U*_*c*_ reaching the onshore crest half period, *T*_*ac*_, but has much more time to develop before *U*_*t*_ reaching the offshore trough half period, *T*_*at*_. The onshore *U*_*B*_ is larger than that offshore with relatively smaller *δ*_*B*_, as described in Eq (11) in sawtooth waves. With the increment in *β*, onshore *δ*_*B*_ decreases and offshore *δ*_*B*_ increases, thereby leading to a larger difference between onshore and offshore *δ*_*B*_ values. Thus, the onshore shear stress is larger than that offshore [11] as observed by Suntoyo et al. [47] and is included in *f* defined by Eq (13). In addition, large amount of offshore picked-up sediments are transported during the onshore duration due to short *T*_*dt*_ [12]. In onshore duration, Δ is larger, and more sediment is carried up than that in offshore duration. Eqs (11) and (13) are important reasons for the correct prediction of onshore net transport rate. The effect of acceleration is the contribution of the boundary layer thickness and phase-lag effect.

The formula of Ribberink [28] also follows *U* well [Fig 9], but cannot predict the difference between onshore and offshore durations without the boundary layer effect. The formula of Nielsen [29] shows the significant difference between onshore and offshore durations by considering shear stress asymmetry, and the difference is consistent with the experiments enlarged by an increment of *β*. But in the said Nielsen formula, *ϕ* unrealistically increases when *D* increases because of increased *f*. In the onshore duration, *ϕ/ϕ*_*m*_ can be approximated by (*U*/*U*_*m*_)^*n*^ [Fig 10]. In the offshore duration, it should be *ϕ/ϕ*_*t*_ because trough rate is relatively decreased by the boundary layer effect. The corresponding *n* in Fig 10a and 10b is larger than that in Fig 5a and 5b, because the phase-lag is smaller with a weaker *U*_*m*_ and larger *D*. The onshore *ϕ/ϕ*_*m*_ of the present fine S556015f (*α*_1_ = 0.67) and S706015f (*α*_1_ = 0.61) can be approximated by *n* = 1.5>1 [Fig 10a and 10b], and the S556015c (*α*_1_ = 0.07) and S706015c (*α*_1_ = 0.04) are *n*≥3 [Fig 10c and 10d].

**Fig 11:**
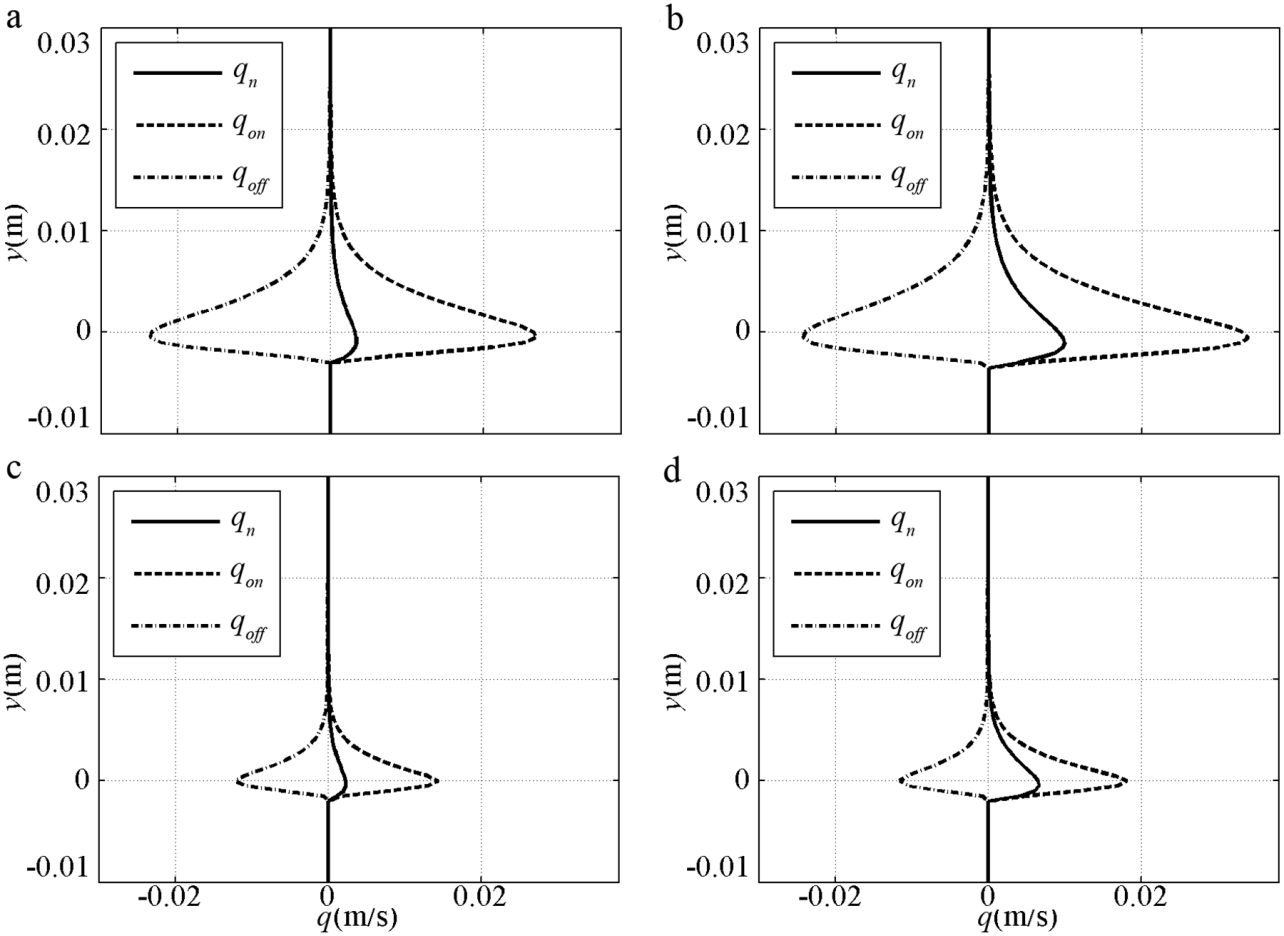
Predicted sediment flux for pure acceleration-skewed flow with *U*_*m*_ = 1.3m/s. a) S556015f: *D* = 0.15mm, *β* = 0.58; b) S706015f: *D* = 0.15mm, *β* = 0.70; c) S556015c: *D* = 0.46mm, *β* = 0.58; d) S706015c: *D* = 0.46mm, *β* = 0.71.

The time-averaged sediment flux of pure acceleration-skewed flow is shown in Fig 11. The total *q*_*on*_, *q*_*off*_ and *q*_*n*_ decrease with increasing *D* and decreasing *β* under the same *U*_*m*_ and *T* conditions. Sediment transport is also mainly generated near the initial bed, but the details are different from those in Fig 6. For the fine cases [Fig 11a and 11b], *q*_*n*_ is onshore but not offshore as that in Fig 6a and 6b. For the coarse cases [Fig 11c and 11d], the bottom level difference between *q*_*on*_ and *q*_*off*_ is not obviously large, which is similar to the experiments in Ruessink et al. [14]. The amount of offshore picked-up sediments that transported during the onshore duration would be not very large in these cases. The onshore net sediment transport rate still exist in pure acceleration-skewed flow below the phase-lag trigger condition of Ω_c_* = 0 or Ω_t_* = 0, which is also seen in the van der A et al.’s [12] and Watanabe and Sato’s [11] experiments with *D*>0.2mm, *U*_*m*_<1m/s, *T*>5s and *β*<0.6. With a relatively large *U*_*B*_ and shear stress in onshore duration due to short time-developed small *δ*_*B*_, *q*_*on*_ is always larger than *q*_*off*_ at every elevation, thereby causing onshore *q*_*n*_ to be in agreement with the onshore net *ϕ* illustrated in Figs 9–10.

**Fig 12:**
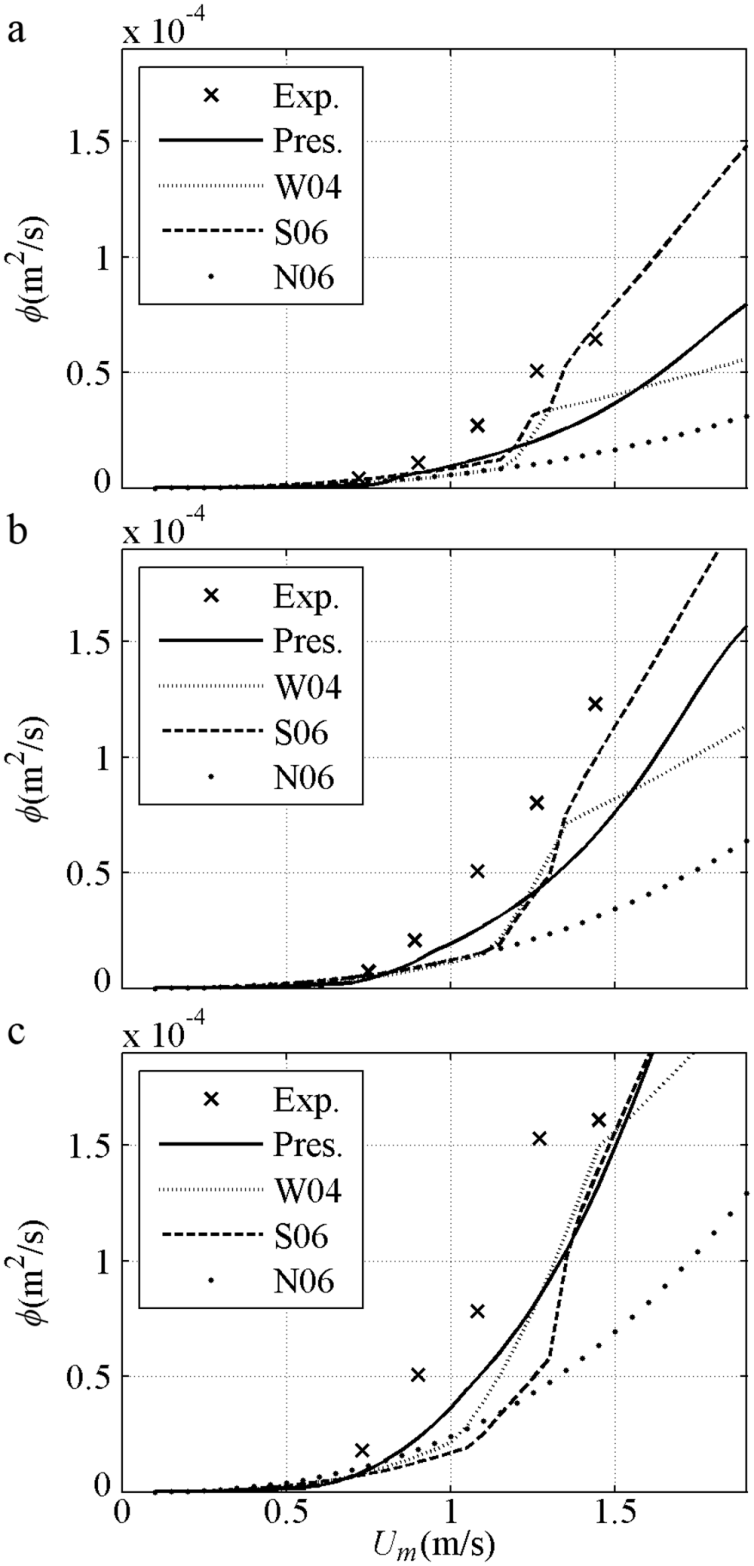
Validation of net sediment transport rate against *U*_*m*_ for pure acceleration-skewed flow. a) *T* = 3s, *D* = 0.2mm, *β* = 0.55; b) *T* = 3s, *D* = 0.2mm, *β* = 0.60; c) *T* = 3s, *D* = 0.2mm, *β* = 0.68.

The net sediment transport rates against *U*_*m*_ for pure acceleration-skewed flows compared with the experiments [11, 12] are validated in Fig 12. The predicted net *ϕ* is onshore, increasing monotonously with an increment in *U*_*m*_ in all models and agreeing with the experimental results. For pure acceleration-skewed flows, the onshore net *ϕ* can be attributed to phase-lag and boundary layer asymmetry, as suggested by Watanabe and Sato [11] with a velocity leaning index and Silva et al. [25] with the wave friction factor in Eq (13). The onshore net *ϕ* is increased by increased phase-lag and *δ*_*B*_ asymmetry with an increment in *β* in Fig 12. The onshore net *ϕ* is only caused by the boundary layer asymmetry below phase-lag trigger condition of Ω_t_* = 0. As shown in Fig 12c in Watanabe and Sato [11], Ω_t_*>0 is initially triggered by the short *T*_*td*_ at *U*_*m*_ = 1.02m/s, thereby leading to a fast-increasing net *ϕ*. The sediment amount to be carried in the *T*_*c*_ duration suddenly becomes unrealistically constant when Ω_t_*>0 until *U*_*m*_ is sufficiently large (1.45m/s) to generate Ω_c_*>0 and a slow-increasing net *ϕ*.

**Fig 13:**
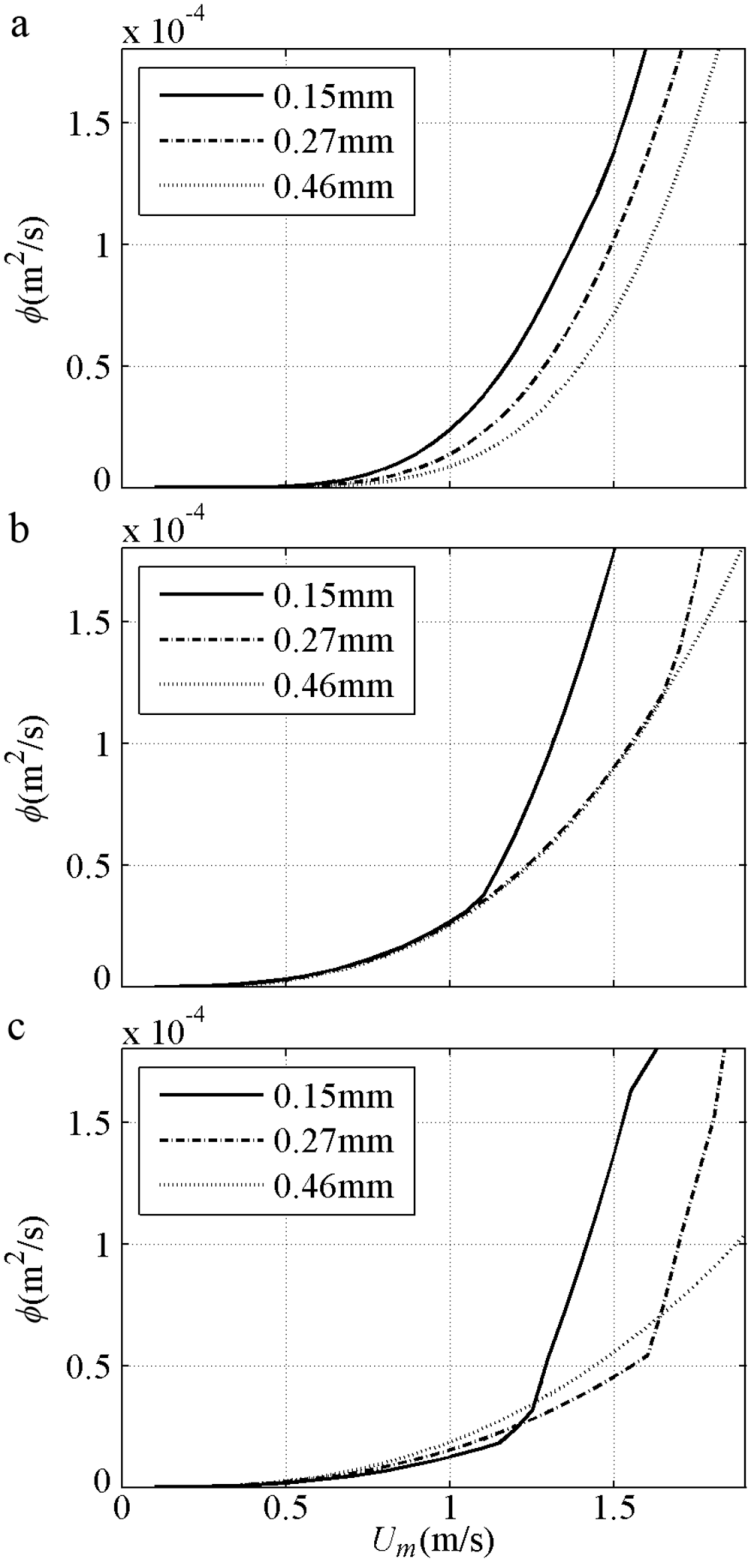
Net sediment transport of Sawtooth flows grouped by *D* with *T* = 6s and *β* = 0.70. a) Present model; b) Watanabe and Sato (2004); c) Silva et al. (2006).

The sediment size effect in net *ϕ* is shown in Fig 13 with *T* = 6s and *β* = 0.70 [12]. In the present model, the net *ϕ* decreases with increasing *D* for the same *U*_*m*_ because Δ is decreased realistically; as a result, *q*_*on*_, *q*_*off*_ and *q*_*n*_ decrease proportionally [Fig 11]. In Watanabe and Sato [11] and Silva et al. [25], the rate of *D* = 0.15mm for the same *U*_*m*_ is the largest after the phase-lag is triggered by Ω_t_*>0 at about *U*_*m*_ = 1.1m/s, and the net *ϕ* of *D* = 0.27mm is larger than that of *D* = 0.46mm also after the phase-lag triggered by the respective Ω_t_*>0 at about *U*_*m*_ = 1.7m/s. Notably, the real phase-lag appears continuously instead of being triggered suddenly, as observed minimum Δ = 3.8*D* in a weaker sinusoidal flow (*U*_*m*_ = 1.26m/s<1.7m/s, *T* = 7.5s and *D* = 0.27mm) by O’Donoghue and Wright [9].

### 3.3 Mixed velocity-skewed and acceleration-skewed flow

**Fig 14:**
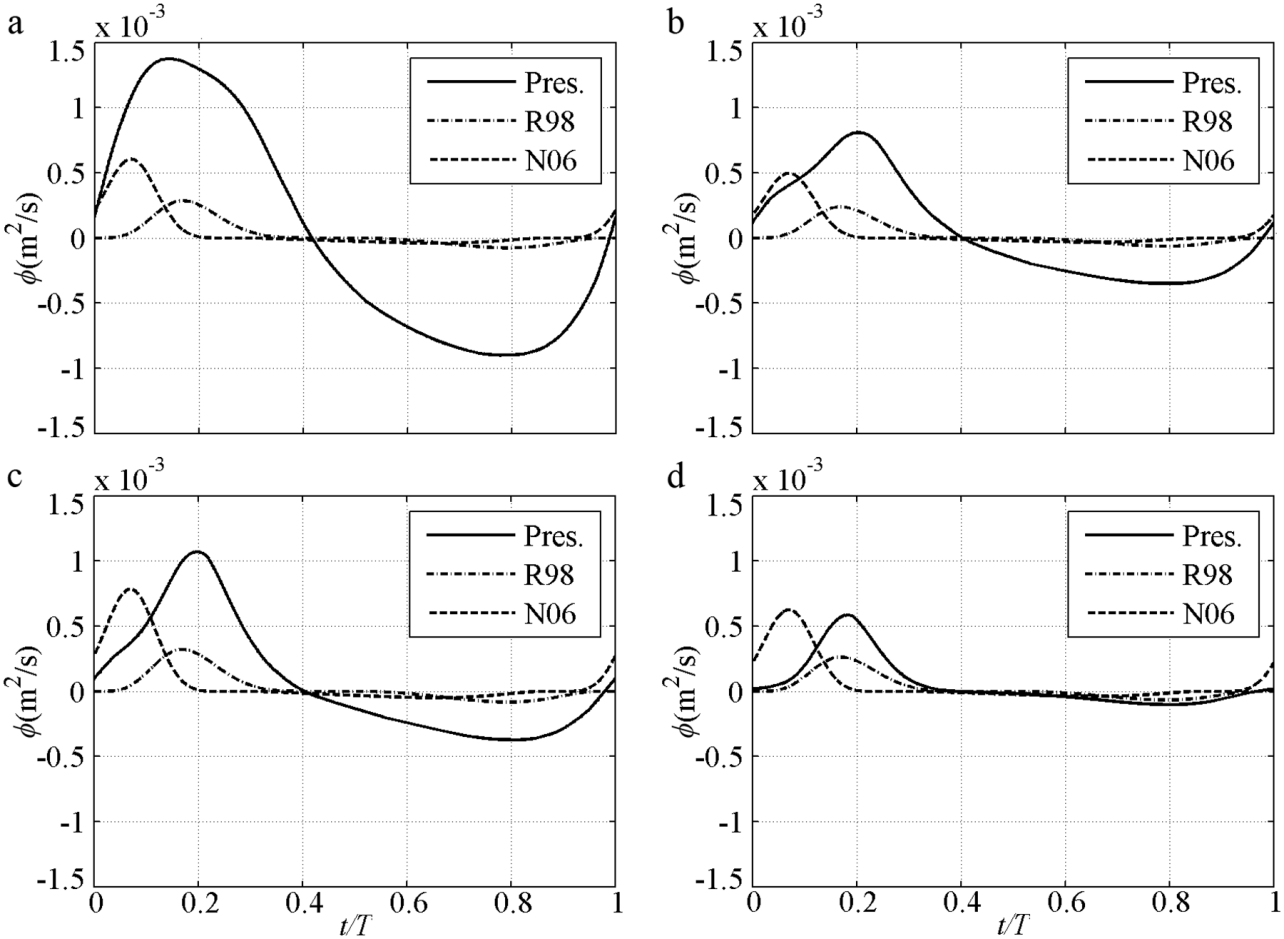
Instantaneous sediment transport rate for mixed flow with *U*_*m*_ = 1.2m/s, *R* = 0.6 and *β* = 0.65. a) W1: *T* = 3s, *D* = 0.16mm, *R* = 0.6; *β* = 0.65; b) W11: *T* = 5s, *D* = 0.16mm, *R* = 0.6; *β* = 0.65; c) W23: *T* = 3s, *D* = 0.3mm, *R* = 0.6; *β* = 0.65; d) W24: *T* = 5s, *D* = 3mm, *R* = 0.6; *β* = 0.65.

**Fig 15:**
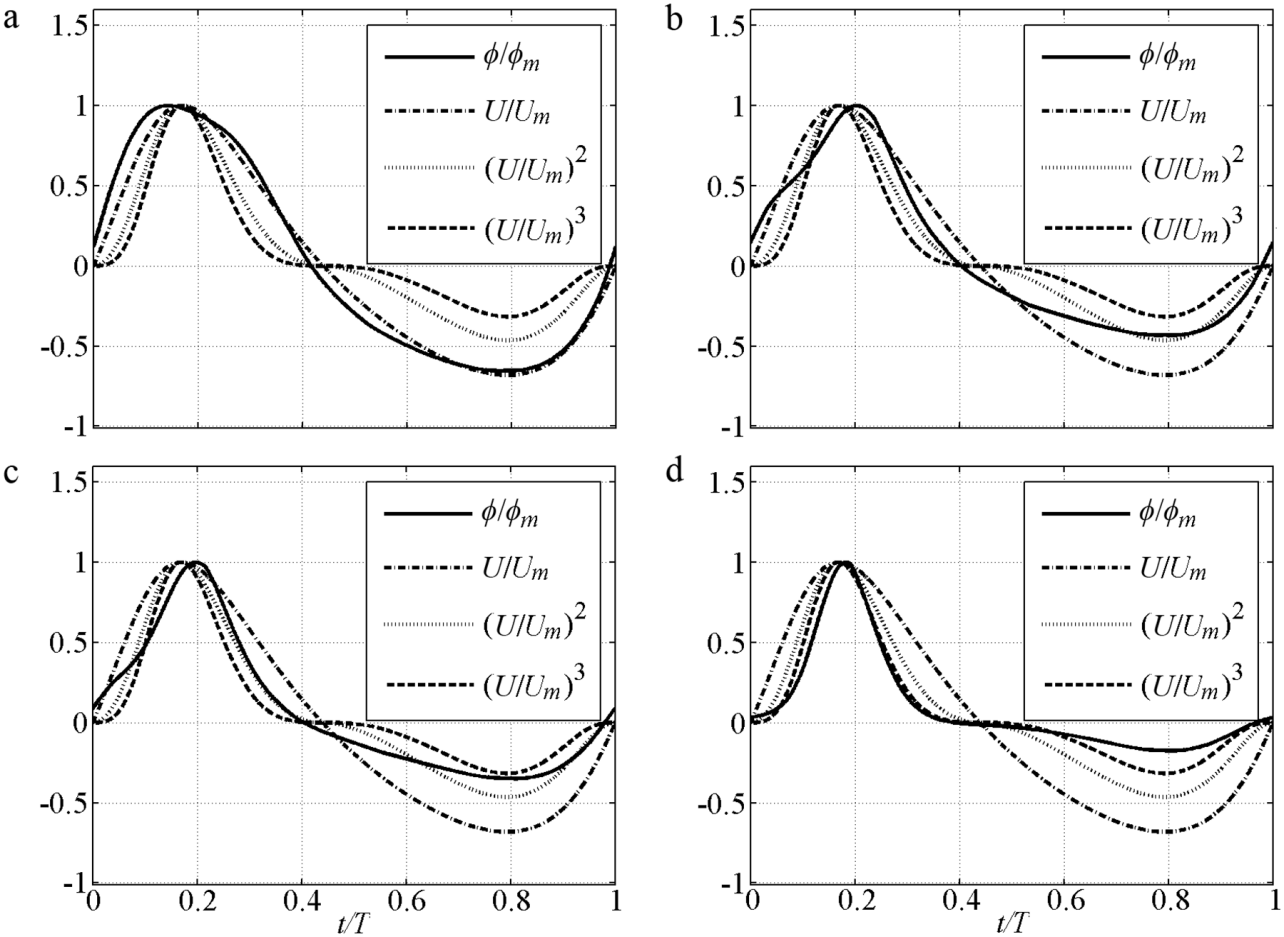
Comparison instantaneous transport rate with *U* for mixed flow with *U*_*m*_ = 1.2m/s, *R* = 0.6 and *β* = 0.65, (*U*/*U*_*m*_)^2^ at offshore stage is mirrored opposite. a) W1: *T* = 3s, *D* = 0.16mm, *R* = 0.6; *β* = 0.65; b) W11: *T* = 5s, *D* = 0.16mm, *R* = 0.6; *β* = 0.65; c) W23: *T* = 3s, *D* = 0.3mm, *R* = 0.6; *β* = 0.65; d) W24: *T* = 5s, *D* = 3mm, *R* = 0.6; *β* = 0.65.

The cases of *U*_*m*_ = *U*_*c*_ = 1.2m/s, *U*_*t*_ = 0.8m/s, *T* = 3.0–5.0s, *D* = 0.16–0.30mm, *R* = 0.6 and *β* = 0.65 [15] are selected considering most *R* = 0.5–0.7 and *β* = 0.5–0.75. The instantaneous *ϕ* is shown in Fig 14, and a comparison of the (*U*/*U*_*m*_)^*n*^ with the present model is provided in Fig 15. The *ϕ* variation is slightly related to *D* in the formulas without suspension sediment of Ribberink [28] and Nielsen [29]. Their onshore *ϕ* is significantly larger than that offshore because *U*_*c*_>*U*_*t*_, as shown in Fig 4 without phase-lag. The *ϕ* decrement with increased *D* is evident in the present model. This result is consistent with Figs 4 and 9 because suspended sediment is considered. The present onshore duration in Fig 15 can be approximated by (*U/U*_*m*_)^*n*^ in cases W1 (*α*_1_ = 0.81) with *n* = 1, W11 (*α*_1_ = 0.62) with *n* = 1.6, W23 (*α*_1_ = 0.50) with *n* = 2 and W24 (*α*_1_ = 0.21) with *n* = 3. In the offshore duration *n* should not be the same as the onshore because the rate at the flow trough is relatively decreased by large *δ*_*B*_ development with long *T*_*at*_, as illustrated in Figs 9–10.

**Fig 16:**
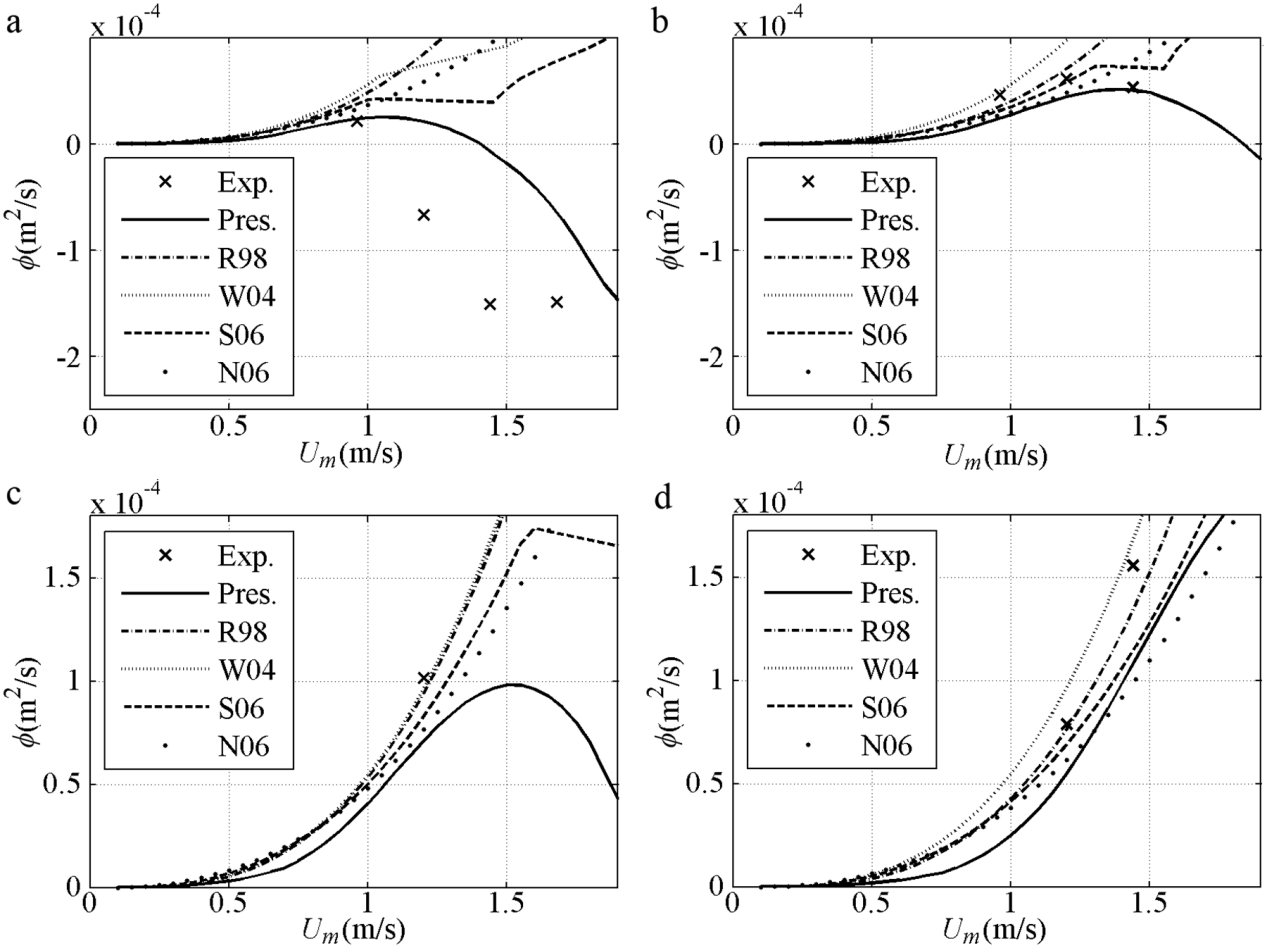
Validation of net sediment transport rate against *U*_*m*_ for mixed flow. a) *T* = 3s, *D* = 0.16mm, *R* = 0.6; *β* = 0.65; b) *T* = 5s, *D* = 0.16mm, *R* = 0.6; *β* = 0.65; c) *T* = 3s, *D* = 0.3mm, *R* = 0.6; *β* = 0.65; d) *T* = 5s, *D* = 3mm, *R* = 0.6; *β* = 0.65.

Following the instantaneous process (Figs 14–15), the net *ϕ* against *U*_*m*_ are shown in Fig 16. In Fig 16a, the experimental results are similar to those in Fig 7c and show a variation from onshore to offshore by increasing *U*_*m*_ with the *T* = 3s and *R* = 0.6. The offshore *ϕ* is relatively stronger than Fig 7c because of the larger phase-lag caused by a smaller *D* (0.16mm<0.2mm), even *U*_*B*_ is increased at the crest and decreased at the trough by *β* = 0.65>0.5. Only the present model can predict the offshore net *ϕ* affected by phase-lag and boundary layer asymmetry, as illustration for Fig 4. In the formulas of Watanabe and Sato [11] and Silver et al. [25], phase-lag is triggered at approximately *U*_*m*_ = 1.0m/s by Ω_c_*>0; however, the net *ϕ* is not decreased to offshore with *β* = 0.65. After about *U*_*m*_ = 1.5m/s, the onshore increment is again enhanced because the offshore sediment amount is suddenly restricted by triggering Ω_t_*>0. In Fig 16b, the three pieces of data are onshore with a smaller phase-lag due to a *T* = 5s>3s in Fig 16a. The results of the Silver et al. [25] and the present model are closer to the actual variation in the three pieces of data than the results of the other formulas. In Fig 16c and 16d, the experimental results can be predicted by all the formulas with larger *D* (0.3mm). Generally, for this type of mixed flow (*R* = 0.6 and *β* = 0.65), the offshore transport tendency is obtained using the present model when the *U*_*m*_ is sufficiently large because the computed onshore *δ*_*B*_ increases faster than offshore *δ*_*B*_.

**Fig 17:**
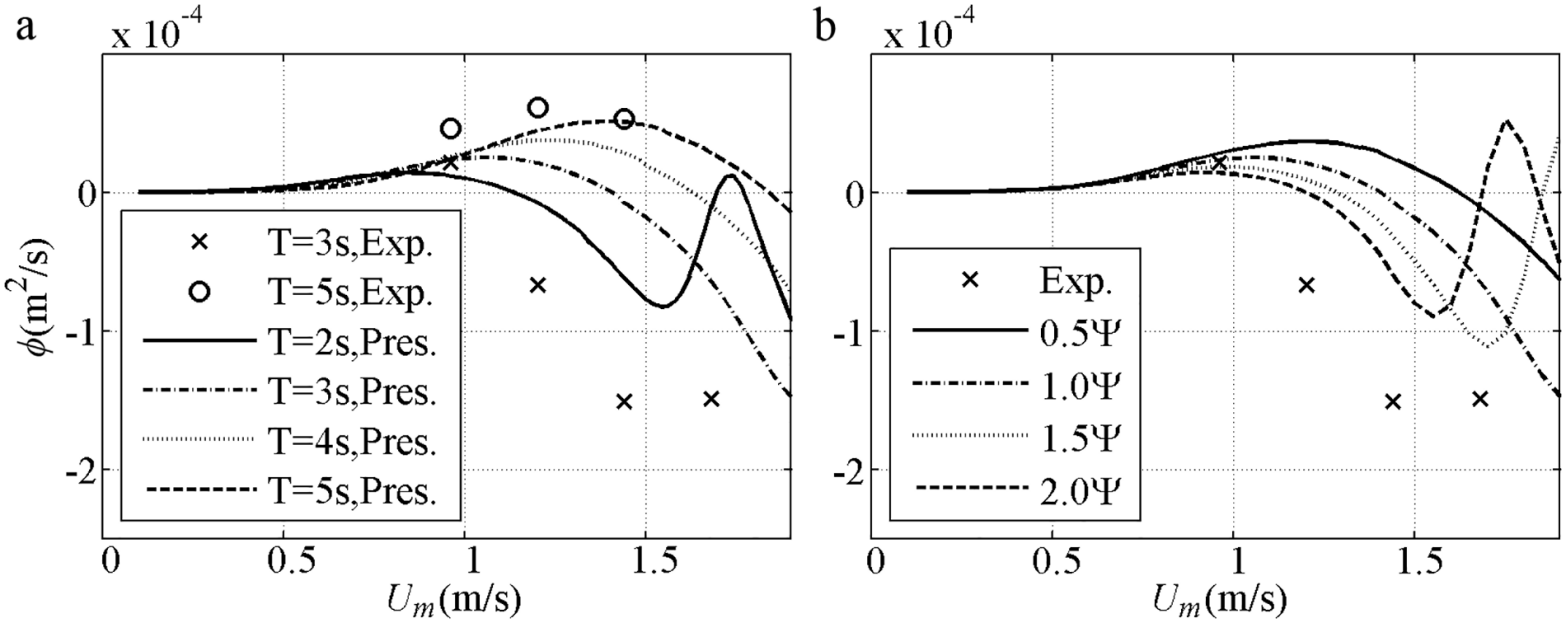
Effect of phase-shift in net sediment transport prediction (*D* = 0.16mm, *R* = 0.6, *β* = 0.65). a) Effect of *T*, *ψ* = Ψ; b) Effect of *ψ*, *T* = 3s.

**Fig 18:**
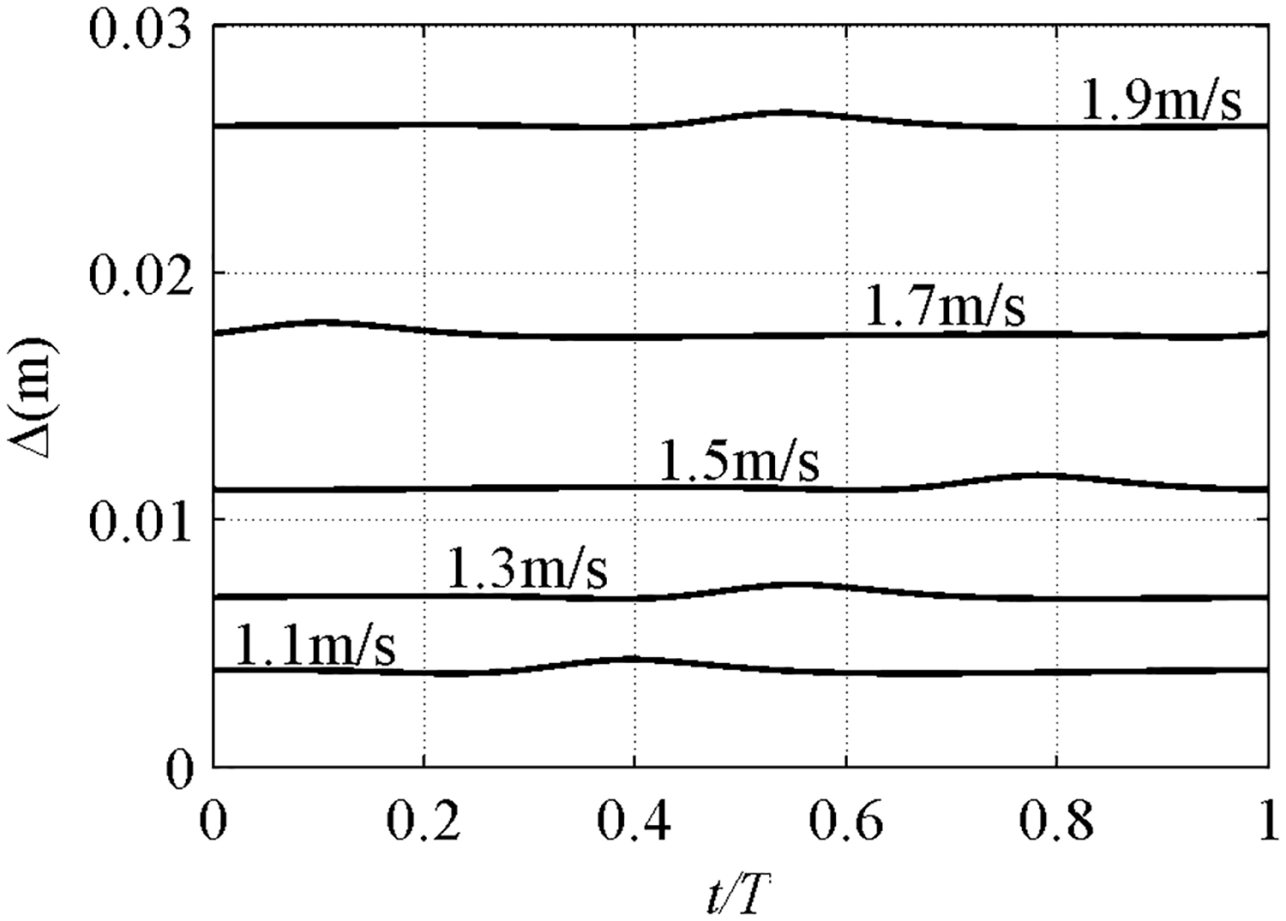
Phase-shift in Δ with *T* = 2s, *D* = 0.16mm, *R* = 0.6, *β* = 0.65, *U*_*m*_ = [1.1,1.3,1.5,1.7,1.9]m/s.

In the previous study, the phase-residual *α*_1_ is vital in the net sediment transport direction, but the effect of phase-shift *ψ* is not clearly shown. The net *ϕ* of *D* = 0.16mm, *T* = 2–5s, *R* = 0.6 and *β* = 0.65 is shown in Fig 17a. With the decrement in *T* from 5s to 2s, both *α*_1_ and *ψ* are increased, thereby leading to the offshore net *ϕ* with a smaller *U*_*m*_. For *T* = 2s, a wavy variation process is caused by the fast increase in *ψ*, as demonstrated by Δ that corresponds to *U*_*m*_ = 1.1–1.9m/s [Fig 18]. From *U*_*m*_ = 1.1 to 1.5m/s (*ψ* = 0.42π to 1.20π), Δ_*m*_ is shifted from *t*/*T* = 0.39 to 0.78, which nearly corresponds to the flow reversal to the trough, thereby leading to more sediments carried by offshore velocity that enhances offshore net *ϕ*. The considerable phase-shift is the reason for large offshore net *ϕ* observed by Dibajnia [6] in 1^st^ Cnoidal flow experiment wherein *T* = 1–1.5s, *D* = 0.2mm and *U*_*m*_<1m/s. At an increment of *U*_*m*_ = 1.7–1.9m/s, Δ_*m*_ is shifted next to onshore–offshore, thereby leading to more sediment carried by onshore–offshore velocity to enhance onshore–offshore net *ϕ* and thus resulting in a wavy variation. In the present model, a full wavy process is shown until *ψ* becomes extremely large to a period (*ψ*>2π, *D* = 0.16mm, *T* = 2s, *U*_*m*_>1.9m/s, *α*_1_>0.97). In the experiment where *T* = 3s, if *ψ* in Eq (14) is set to 1.5Ψ–2.0Ψ, a wavy process is also seen [Fig 17b].

### 3.4 Net sediment transport rates

**Fig 19:**
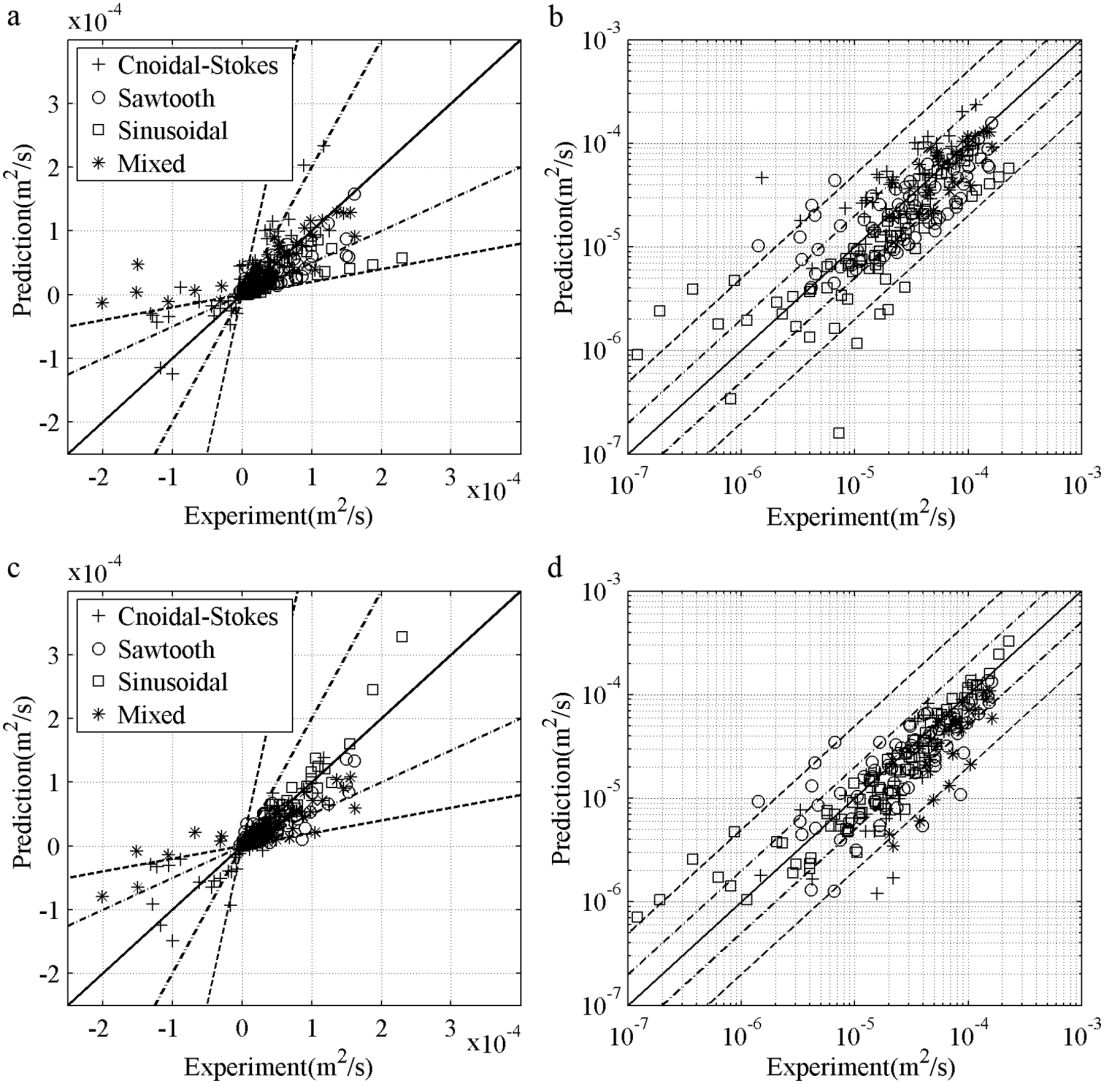
Net sediment transport rate validation. a) View of Cartesian coordinates, van der A et al. [32]; b) View of log-log coordinates, van der A et al. [32]; c) View of Cartesian coordinates, Eq (7); d) View of log-log coordinates, Eq (7).

The net *ϕ* error can be easily enlarged by the cumulative integration of the instantaneous process. As shown in Fig 9c, the formula of Ribberink [28] is significantly close to the present model for pure acceleration-skewed case S556015c, but the cumulative integration is always zero. The validity of Eq (7) is compared to van der A et al.’s [32] formula with data in Table 1, including half-period sinusoidal flows, pure velocity-skewed flows (1^st^ Cnoidal and 2^nd^ Stokes flows), pure acceleration-skewed flows (Sawtooth flows) and mixed velocity- and acceleration-skewed flows. The results grouped by wave shapes are shown in Fig 19, where the solid line represents the accurate prediction, the dotted lines represent the twofold deviation, and the dashed lines represent the fivefold deviation. The onshore data are too crowded to be recognised, so a log–log view is also shown. The offshore net *ϕ* are observed in velocity-skewed flows, which have large phase-lag, as fine *D* [10, 43, 44], short *T* [6] or large *U*_*m*_ [8, 15], and can be predicted by Eq (7) with continuously varying wave boundary layer thickness and phase-lag. The net *ϕ* of the pure acceleration-skewed flows are always onshore [11–12], and can be satisfactorily predicted by accounting for acceleration reflected by boundary layer asymmetry. The errors are shown in Table 2, where PD, P2 and P5 denote the data percentage within the correct direction, twofold deviation and fivefold deviation respectively. The prediction obtained by the present model agrees with the experimental results well in magnitude and direction with good percentage data in the specific deviations (P2 = 71.31%, P5 = 90.16%, PD = 97.13%) that are slightly better than van der A et al. [32]. This would be realistic because in van der A et al.’s [32] the phase-lag is triggered suddenly and net current caused by asymmetric boundary layer development is not contained. These results further prove the importance of comprehensive considering suspended sediment, continuous phase-lag and asymmetric boundary layer development.

**Table 2:** Net sediment transport rate prediction errors.

Models	P2(%)	P5(%)	PD(%)
Equation (7)	71.31	90.16	97.13
van der A et al. [32]	64.34	87.29	95.90

## 4. Conclusions

An instantaneous analytical model for asymmetric oscillatory sheet flow sediment transport is proposed by integrating concentration profile and velocity profile. The concentration profile is approximated by the exponential law based on mass conservation, and the asymmetric velocity profile with a phase-ahead near the initial bed to the free stream velocity is derived by boundary flow theory [35] and asymmetric wave theory [34]. The model takes into account the suspended sediment, phase-lag and asymmetric boundary layer development, and can reduce to the classical 3/2 power law in terms of the Shields parameter.

Instantaneous sediment transport is separately studied for the pure velocity-skewed flows, the pure acceleration-skewed flows, and the mixed velocity-skewed and acceleration-skewed flows. Different exponents of velocity power function in the instantaneous transport rate are unified, summarised and validated as exponents decrease with increment in phase-lag. The decrease tendency of the instantaneous transport rate to increment in sediment size under the same flow conditions is well predicted by the present model because erosion depth contains suspended sediment. Also, the model can provide the similar result to the two-phase model.

The present model is the only instantaneous model that can predict the net sediment transport rate in asymmetric oscillatory sheet flows. In the pure velocity-skewed flows, onshore net sediment transport rate is shown generated when phase-lag is relatively small, because of the recognised significant erosion depth difference between onshore and offshore; while large offshore net sediment transport rate is shown generated when phase-lag is large, because of the relatively large offshore velocity in boundary layer represented by the relatively small offshore wave boundary layer thickness. In the pure acceleration-skewed flows, if an acceleration skewness degree *β*>0.5, the net sediment transport rate is shown onshore and increasing with increased *β* by the present model. One reason is the consideration of the asymmetric boundary layer development, and the other reason is the phase-lag. Over all, the phase-lag and the asymmetric boundary layer development are shown both necessary for the net sediment transport in asymmetric oscillatory sheet flows.

## Appendix

### Notations

*A* the oscillatory flow orbital amplitude;
*D* the sediment diameter;
*f* the wave friction factor
*g* the gravitational acceleration;
*i* the imaginary unit;
*k*_*N*_ the roughness height over a mobile bed;
*n* the exponent of power function;
*R* velocity asymmetry parameter;
*S* the volumetric concentration;
*s* the sediment specific gravity;
*T* the period;
*t* the time;
*U* Im(**W**);
*U*_*k*_ the *k*^th^ harmonic velocity amplitude;
*V* Re(**W**);
**W** the complex free stream velocity;
**W**_*B*_ the complex velocity in the wave boundary layer;
*w* the sediment falling velocity;
*y* the vertical coordinate;
*α* the phase-lead parameter;
*α*_1_ the phase-residual;
*α*_2_ a parameter denotes the periodic variation of erosion depth;
*α*_Δ_ a parameter equal Δ_*m*_*/U*_*m*_^2^;
*β* the acceleration degree;
Δ the erosion depth;
*δ* a parameter given by the turbulent wave boundary layer thickness;
*δ*_*B*_ the turbulent wave boundary layer thickness;
*δ*_*S*_ the maximum sheet flow layer thickness;
Θ the Shields parameter;
*ϕ* the sediment transport rate;
Φ the dimensionless *ϕ*;
*φ*_*k*_ the wave form parameter;
Ψ the phase-lag parameter;
*ψ* the phase-shift;
*ω* the angular frequency.

### Subscripts

*a* the acceleration duration;
*B* the boundary layer;
*c* the flow crest duration;
*d* the deceleration duration;
*k* serial number;
*m* the maximum value;
*rc* the flow reversal after flow crest;
*rt* the flow reversal after flow trough;
*t* the flow trough duration.

## Supporting information

S1 Data Data_underlying_finding. (DOCX)
