# Supplementary material for: Instantaneous sediment transport model for asymmetric oscillatory sheet flow
Source: PLoS One. 2017 Dec 22;12(12):e0190034. doi: 10.1371/journal.pone.0190034 (PMC5741249; doi:10.1371/journal.pone.0190034)
Supplement: S1 Data — (DOCX) [file pone.0190034.s001.docx]

**Data underlying finding**

Figure 1.

*t*=[0.00E+00 1.00E-02 2.00E-02 3.00E-02 4.00E-02 5.00E-02 6.00E-02 7.00E-02 8.00E-02 9.00E-02 1.00E-01 1.10E-01 1.20E-01 1.30E-01 1.40E-01 1.50E-01 1.60E-01 1.70E-01 1.80E-01 1.90E-01 2.00E-01 2.10E-01 2.20E-01 2.30E-01 2.40E-01 2.50E-01 2.60E-01 2.70E-01 2.80E-01 2.90E-01 3.00E-01 3.10E-01 3.20E-01 3.30E-01 3.40E-01 3.50E-01 3.60E-01 3.70E-01 3.80E-01 3.90E-01 4.00E-01 4.10E-01 4.20E-01 4.30E-01 4.40E-01 4.50E-01 4.60E-01 4.70E-01 4.80E-01 4.90E-01 5.00E-01 5.10E-01 5.20E-01 5.30E-01 5.40E-01 5.50E-01 5.60E-01 5.70E-01 5.80E-01 5.90E-01 6.00E-01 6.10E-01 6.20E-01 6.30E-01 6.40E-01 6.50E-01 6.60E-01 6.70E-01 6.80E-01 6.90E-01 7.00E-01 7.10E-01 7.20E-01 7.30E-01 7.40E-01 7.50E-01 7.60E-01 7.70E-01 7.80E-01 7.90E-01 8.00E-01 8.10E-01 8.20E-01 8.30E-01 8.40E-01 8.50E-01 8.60E-01 8.70E-01 8.80E-01 8.90E-01 9.00E-01 9.10E-01 9.20E-01 9.30E-01 9.40E-01 9.50E-01 9.60E-01 9.70E-01 9.80E-01 9.90E-01 1.00E+00];

*U*=[0.00E+00 9.00E-02 1.87E-01 2.86E-01 3.86E-01 4.87E-01 5.86E-01 6.82E-01 7.72E-01 8.57E-01 9.34E-01 1.00E+00 1.06E+00 1.11E+00 1.14E+00 1.17E+00 1.18E+00 1.19E+00 1.18E+00 1.17E+00 1.15E+00 1.12E+00 1.09E+00 1.05E+00 1.00E+00 9.55E-01 9.05E-01 8.53E-01 8.00E-01 7.46E-01 6.91E-01 6.37E-01 5.82E-01 5.28E-01 4.75E-01 4.22E-01 3.71E-01 3.20E-01 2.70E-01 2.22E-01 1.75E-01 1.29E-01 8.44E-02 4.09E-02 -1.41E-03 -4.25E-02 -8.24E-02 -1.21E-01 -1.59E-01 -1.95E-01 -2.31E-01 -2.65E-01 -2.99E-01 -3.31E-01 -3.62E-01 -3.93E-01 -4.22E-01 -4.51E-01 -4.78E-01 -5.05E-01 -5.30E-01 -5.55E-01 -5.79E-01 -6.02E-01 -6.24E-01 -6.44E-01 -6.64E-01 -6.83E-01 -7.01E-01 -7.18E-01 -7.33E-01 -7.48E-01 -7.61E-01 -7.73E-01 -7.83E-01 -7.92E-01 -7.99E-01 -8.05E-01 -8.09E-01 -8.10E-01 -8.10E-01 -8.07E-01 -8.02E-01 -7.94E-01 -7.83E-01 -7.69E-01 -7.51E-01 -7.30E-01 -7.05E-01 -6.76E-01 -6.41E-01 -6.03E-01 -5.58E-01 -5.09E-01 -4.54E-01 -3.93E-01 -3.26E-01 -2.54E-01 -1.76E-01 -9.18E-02 0.00E+00];

Figure 2.

*t/T*=[0.00E+00 1.00E-02 2.00E-02 3.00E-02 4.00E-02 5.00E-02 6.00E-02 7.00E-02 8.00E-02 9.00E-02 1.00E-01 1.10E-01 1.20E-01 1.30E-01 1.40E-01 1.50E-01 1.60E-01 1.70E-01 1.80E-01 1.90E-01 2.00E-01 2.10E-01 2.20E-01 2.30E-01 2.40E-01 2.50E-01 2.60E-01 2.70E-01 2.80E-01 2.90E-01 3.00E-01 3.10E-01 3.20E-01 3.30E-01 3.40E-01 3.50E-01 3.60E-01 3.70E-01 3.80E-01 3.90E-01 4.00E-01 4.10E-01 4.20E-01 4.30E-01 4.40E-01 4.50E-01 4.60E-01 4.70E-01 4.80E-01 4.90E-01 5.00E-01 5.10E-01 5.20E-01 5.30E-01 5.40E-01 5.50E-01 5.60E-01 5.70E-01 5.80E-01 5.90E-01 6.00E-01 6.10E-01 6.20E-01 6.30E-01 6.40E-01 6.50E-01 6.60E-01 6.70E-01 6.80E-01 6.90E-01 7.00E-01 7.10E-01 7.20E-01 7.30E-01 7.40E-01 7.50E-01 7.60E-01 7.70E-01 7.80E-01 7.90E-01 8.00E-01 8.10E-01 8.20E-01 8.30E-01 8.40E-01 8.50E-01 8.60E-01 8.70E-01 8.80E-01 8.90E-01 9.00E-01 9.10E-01 9.20E-01 9.30E-01 9.40E-01 9.50E-01 9.60E-01 9.70E-01 9.80E-01 9.90E-01 1.00E+00];

(1) With phase-lead and phase-shift; *ϕ*/*ϕ_m_*: 0.5m/s, 1.0m/s, 1.5m/s, 2.0m/s

[8.89E-04 8.94E-04 1.23E-03 2.18E-03 4.26E-03 8.38E-03 1.57E-02 2.78E-02 4.64E-02 7.31E-02 1.09E-01 1.53E-01 2.06E-01 2.67E-01 3.37E-01 4.14E-01 4.95E-01 5.79E-01 6.63E-01 7.43E-01 8.17E-01 8.81E-01 9.33E-01 9.72E-01 9.94E-01 1.00E+00 9.88E-01 9.60E-01 9.16E-01 8.58E-01 7.90E-01 7.12E-01 6.29E-01 5.43E-01 4.58E-01 3.77E-01 3.01E-01 2.33E-01 1.74E-01 1.24E-01 8.37E-02 5.14E-02 2.87E-02 1.39E-02 5.13E-03 5.84E-04 -1.27E-03 -1.66E-03 -1.44E-03 -1.11E-03 -8.89E-04 -8.94E-04 -1.23E-03 -2.18E-03 -4.26E-03 -8.38E-03 -1.57E-02 -2.78E-02 -4.64E-02 -7.31E-02 -1.09E-01 -1.53E-01 -2.06E-01 -2.67E-01 -3.37E-01 -4.14E-01 -4.95E-01 -5.79E-01 -6.63E-01 -7.43E-01 -8.17E-01 -8.81E-01 -9.33E-01 -9.72E-01 -9.94E-01 -1.00E+0 -9.88E-01 -9.60E-01 -9.16E-01 -8.58E-01 -7.90E-01 -7.12E-01 -6.29E-01 -5.43E-01 -4.58E-01 -3.77E-01 -3.01E-01 -2.33E-01 -1.74E-01 -1.24E-01 -8.37E-02 -5.14E-02 -2.87E-02 -1.39E-02 -5.13E-03 -5.84E-04 1.27E-03 1.66E-03 1.44E-03 1.11E-03 8.89E-04]

[1.36E-01 1.64E-01 1.90E-01 2.15E-01 2.40E-01 2.64E-01 2.79E-01 2.96E-01 3.17E-01 3.41E-01 3.68E-01 4.00E-01 4.35E-01 4.74E-01 5.17E-01 5.63E-01 6.11E-01 6.61E-01 7.12E-01 7.62E-01 8.10E-01 8.56E-01 8.97E-01 9.33E-01 9.62E-01 9.83E-01 9.96E-01 1.00E+00 9.94E-01 9.78E-01 9.53E-01 9.18E-01 8.75E-01 8.25E-01 7.68E-01 7.06E-01 6.40E-01 5.72E-01 5.02E-01 4.33E-01 3.65E-01 3.00E-01 2.37E-01 1.78E-01 1.23E-01 7.14E-02 1.84E-02 -2.87E-02 -6.96E-02 -1.05E-01 -1.36E-01 -1.64E-01 -1.90E-01 -2.15E-01 -2.40E-01 -2.64E-01 -2.79E-01 -2.96E-01 -3.17E-01 -3.41E-01 -3.68E-01 -4.00E-01 -4.35E-01 -4.74E-01 -5.17E-01 -5.63E-01 -6.11E-01 -6.61E-01 -7.12E-01 -7.62E-01 -8.10E-01 -8.56E-01 -8.97E-01 -9.33E-01 -9.62E-01 -9.83E-01 -9.96E-01 -1.00E+0 -9.94E-01 -9.78E-01 -9.53E-01 -9.18E-01 -8.75E-01 -8.25E-01 -7.68E-01 -7.06E-01 -6.40E-01 -5.72E-01 -5.02E-01 -4.33E-01 -3.65E-01 -3.00E-01 -2.37E-01 -1.78E-01 -1.23E-01 -7.14E-02 -1.84E-02 2.87E-02 6.96E-02 1.05E-01 1.36E-01]

[2.23E-01 3.21E-01 4.14E-01 5.01E-01 5.66E-01 6.22E-01 6.70E-01 7.11E-01 7.46E-01 7.76E-01 8.02E-01 8.24E-01 8.44E-01 8.61E-01 8.77E-01 8.91E-01 9.04E-01 9.16E-01 9.28E-01 9.40E-01 9.51E-01 9.61E-01 9.71E-01 9.80E-01 9.88E-01 9.94E-01 9.98E-01 1.00E+00 9.99E-01 9.95E-01 9.87E-01 9.74E-01 9.57E-01 9.35E-01 9.08E-01 8.75E-01 8.36E-01 7.92E-01 7.42E-01 6.87E-01 6.27E-01 5.61E-01 4.91E-01 4.17E-01 3.39E-01 2.58E-01 1.74E-01 8.76E-02 -1.79E-02 -1.22E-01 -2.23E-01 -3.21E-01 -4.14E-01 -5.01E-01 -5.66E-01 -6.22E-01 -6.70E-01 -7.11E-01 -7.46E-01 -7.76E-01 -8.02E-01 -8.24E-01 -8.44E-01 -8.61E-01 -8.77E-01 -8.91E-01 -9.04E-01 -9.16E-01 -9.28E-01 -9.40E-01 -9.51E-01 -9.61E-01 -9.71E-01 -9.80E-01 -9.88E-01 -9.94E-01 -9.98E-01 -1.00E+0 -9.99E-01 -9.95E-01 -9.87E-01 -9.74E-01 -9.57E-01 -9.35E-01 -9.08E-01 -8.75E-01 -8.36E-01 -7.92E-01 -7.42E-01 -6.87E-01 -6.27E-01 -5.61E-01 -4.91E-01 -4.17E-01 -3.39E-01 -2.58E-01 -1.74E-01 -8.76E-02 1.79E-02 1.22E-01 2.23E-01]

[1.13E-01 1.91E-01 2.69E-01 3.44E-01 4.12E-01 4.76E-01 5.36E-01 5.93E-01 6.47E-01 6.97E-01 7.44E-01 7.87E-01 8.27E-01 8.63E-01 8.95E-01 9.22E-01 9.46E-01 9.65E-01 9.80E-01 9.91E-01 9.98E-01 1.00E+00 9.98E-01 9.92E-01 9.83E-01 9.69E-01 9.52E-01 9.32E-01 9.09E-01 8.84E-01 8.55E-01 8.25E-01 7.92E-01 7.57E-01 7.20E-01 6.82E-01 6.42E-01 6.01E-01 5.58E-01 5.14E-01 4.68E-01 4.21E-01 3.72E-01 3.22E-01 2.69E-01 2.15E-01 1.59E-01 1.01E-01 4.07E-02 -3.60E-02 -1.13E-01 -1.91E-01 -2.69E-01 -3.44E-01 -4.12E-01 -4.76E-01 -5.36E-01 -5.93E-01 -6.47E-01 -6.97E-01 -7.44E-01 -7.87E-01 -8.27E-01 -8.63E-01 -8.95E-01 -9.22E-01 -9.46E-01 -9.65E-01 -9.80E-01 -9.91E-01 -9.98E-01 -1.00E+0 -9.98E-01 -9.92E-01 -9.83E-01 -9.69E-01 -9.52E-01 -9.32E-01 -9.09E-01 -8.84E-01 -8.55E-01 -8.25E-01 -7.92E-01 -7.57E-01 -7.20E-01 -6.82E-01 -6.42E-01 -6.01E-01 -5.58E-01 -5.14E-01 -4.68E-01 -4.21E-01 -3.72E-01 -3.22E-01 -2.69E-01 -2.15E-01 -1.59E-01 -1.01E-01 -4.07E-02 3.60E-02 1.13E-01]

(2) Without phase-lead and phase-shift; *ϕ/ϕ_m_*: 0.5m/s, 1.0m/s, 1.5m/s, 2.0m/s

[0.00E+0 1.77E-04 5.50E-04 1.45E-03 3.44E-03 7.42E-03 1.46E-02 2.64E-02 4.45E-02 7.06E-02 1.06E-01 1.49E-01 2.01E-01 2.61E-01 3.30E-01 4.06E-01 4.86E-01 5.70E-01 6.53E-01 7.33E-01 8.07E-01 8.73E-01 9.27E-01 9.67E-01 9.92E-01 1.00E+00 9.92E-01 9.67E-01 9.27E-01 8.73E-01 8.07E-01 7.33E-01 6.53E-01 5.70E-01 4.86E-01 4.06E-01 3.30E-01 2.61E-01 2.01E-01 1.49E-01 1.06E-01 7.06E-02 4.45E-02 2.64E-02 1.46E-02 7.42E-03 3.44E-03 1.45E-03 5.50E-04 1.77E-04 2.90E-19 -1.77E-04 -5.50E-04 -1.45E-03 -3.44E-03 -7.42E-03 -1.46E-02 -2.64E-02 -4.45E-02 -7.06E-02 -1.06E-01 -1.49E-01 -2.01E-01 -2.61E-01 -3.30E-01 -4.06E-01 -4.86E-01 -5.70E-01 -6.53E-01 -7.33E-01 -8.07E-01 -8.73E-01 -9.27E-01 -9.67E-01 -9.92E-01 -1.00E+0 -9.92E-01 -9.67E-01 -9.27E-01 -8.73E-01 -8.07E-01 -7.33E-01 -6.53E-01 -5.70E-01 -4.86E-01 -4.06E-01 -3.30E-01 -2.61E-01 -2.01E-01 -1.49E-01 -1.06E-01 -7.06E-02 -4.45E-02 -2.64E-02 -1.46E-02 -7.42E-03 -3.44E-03 -1.45E-03 -5.50E-04 -1.77E-04 -5.80E-19]

[0.00E+0 2.90E-02 5.90E-02 9.09E-02 1.26E-01 1.63E-01 1.95E-01 2.31E-01 2.70E-01 3.12E-01 3.58E-01 4.08E-01 4.60E-01 5.15E-01 5.72E-01 6.30E-01 6.87E-01 7.43E-01 7.97E-01 8.46E-01 8.90E-01 9.28E-01 9.59E-01 9.82E-01 9.95E-01 1.00E+00 9.95E-01 9.82E-01 9.59E-01 9.28E-01 8.90E-01 8.46E-01 7.97E-01 7.43E-01 6.87E-01 6.30E-01 5.72E-01 5.15E-01 4.60E-01 4.08E-01 3.58E-01 3.12E-01 2.70E-01 2.31E-01 1.95E-01 1.63E-01 1.26E-01 9.09E-02 5.90E-02 2.90E-02 5.63E-17 -2.90E-02 -5.90E-02 -9.09E-02 -1.26E-01 -1.63E-01 -1.95E-01 -2.31E-01 -2.70E-01 -3.12E-01 -3.58E-01 -4.08E-01 -4.60E-01 -5.15E-01 -5.72E-01 -6.30E-01 -6.87E-01 -7.43E-01 -7.97E-01 -8.46E-01 -8.90E-01 -9.28E-01 -9.59E-01 -9.82E-01 -9.95E-01 -1.00E+0 -9.95E-01 -9.82E-01 -9.59E-01 -9.28E-01 -8.90E-01 -8.46E-01 -7.97E-01 -7.43E-01 -6.87E-01 -6.30E-01 -5.72E-01 -5.15E-01 -4.60E-01 -4.08E-01 -3.58E-01 -3.12E-01 -2.70E-01 -2.31E-01 -1.95E-01 -1.63E-01 -1.26E-01 -9.09E-02 -5.90E-02 -2.90E-02 -1.13E-16]

[0.00E+0 6.53E-02 1.31E-01 1.96E-01 2.49E-01 3.01E-01 3.50E-01 3.99E-01 4.48E-01 4.96E-01 5.43E-01 5.90E-01 6.36E-01 6.82E-01 7.25E-01 7.67E-01 8.07E-01 8.44E-01 8.79E-01 9.09E-01 9.36E-01 9.59E-01 9.77E-01 9.89E-01 9.97E-01 1.00E+00 9.97E-01 9.89E-01 9.77E-01 9.59E-01 9.36E-01 9.09E-01 8.79E-01 8.44E-01 8.07E-01 7.67E-01 7.25E-01 6.82E-01 6.36E-01 5.90E-01 5.43E-01 4.96E-01 4.48E-01 3.99E-01 3.50E-01 3.01E-01 2.49E-01 1.96E-01 1.31E-01 6.53E-02 1.27E-16 -6.53E-02 -1.31E-01 -1.96E-01 -2.49E-01 -3.01E-01 -3.50E-01 -3.99E-01 -4.48E-01 -4.96E-01 -5.43E-01 -5.90E-01 -6.36E-01 -6.82E-01 -7.25E-01 -7.67E-01 -8.07E-01 -8.44E-01 -8.79E-01 -9.09E-01 -9.36E-01 -9.59E-01 -9.77E-01 -9.89E-01 -9.97E-01 -1.00E+0 -9.97E-01 -9.89E-01 -9.77E-01 -9.59E-01 -9.36E-01 -9.09E-01 -8.79E-01 -8.44E-01 -8.07E-01 -7.67E-01 -7.25E-01 -6.82E-01 -6.36E-01 -5.90E-01 -5.43E-01 -4.96E-01 -4.48E-01 -3.99E-01 -3.50E-01 -3.01E-01 -2.49E-01 -1.96E-01 -1.31E-01 -6.53E-02 -2.54E-16]

[0.00E+0 7.49E-02 1.50E-01 2.15E-01 2.76E-01 3.34E-01 3.90E-01 4.44E-01 4.96E-01 5.47E-01 5.95E-01 6.42E-01 6.86E-01 7.29E-01 7.69E-01 8.06E-01 8.41E-01 8.73E-01 9.02E-01 9.27E-01 9.49E-01 9.67E-01 9.81E-01 9.92E-01 9.98E-01 1.00E+00 9.98E-01 9.92E-01 9.81E-01 9.67E-01 9.49E-01 9.27E-01 9.02E-01 8.73E-01 8.41E-01 8.06E-01 7.69E-01 7.29E-01 6.86E-01 6.42E-01 5.95E-01 5.47E-01 4.96E-01 4.44E-01 3.90E-01 3.34E-01 2.76E-01 2.15E-01 1.50E-01 7.49E-02 1.46E-16 -7.49E-02 -1.50E-01 -2.15E-01 -2.76E-01 -3.34E-01 -3.90E-01 -4.44E-01 -4.96E-01 -5.47E-01 -5.95E-01 -6.42E-01 -6.86E-01 -7.29E-01 -7.69E-01 -8.06E-01 -8.41E-01 -8.73E-01 -9.02E-01 -9.27E-01 -9.49E-01 -9.67E-01 -9.81E-01 -9.92E-01 -9.98E-01 -1.00E+0 -9.98E-01 -9.92E-01 -9.81E-01 -9.67E-01 -9.49E-01 -9.27E-01 -9.02E-01 -8.73E-01 -8.41E-01 -8.06E-01 -7.69E-01 -7.29E-01 -6.86E-01 -6.42E-01 -5.95E-01 -5.47E-01 -4.96E-01 -4.44E-01 -3.90E-01 -3.34E-01 -2.76E-01 -2.15E-01 -1.50E-01 -7.49E-02 -2.92E-16]

Figure 3.

(1) Pure velocity-skewed flow;

*U/U_m_* and *a/a_m_* are obtained from 2^nd^ Stokes flow of Liu and Sato (2006)

(2) Pure acceleration-skewed flow

*U/U_m_* and *a/a_m_* are from Sawtooth flow of van der A et al (2010a)

Figure 4. ‘Exp’ is collected from O’Donoghue and Wright (2004a, 2004b); ‘R98’ is given by Ribberink (1998); ‘N06’ is given by Nielsen (2006).

*t/T*=[0.00E+00 1.00E-02 2.00E-02 3.00E-02 4.00E-02 5.00E-02 6.00E-02 7.00E-02 8.00E-02 9.00E-02 1.00E-01 1.10E-01 1.20E-01 1.30E-01 1.40E-01 1.50E-01 1.60E-01 1.70E-01 1.80E-01 1.90E-01 2.00E-01 2.10E-01 2.20E-01 2.30E-01 2.40E-01 2.50E-01 2.60E-01 2.70E-01 2.80E-01 2.90E-01 3.00E-01 3.10E-01 3.20E-01 3.30E-01 3.40E-01 3.50E-01 3.60E-01 3.70E-01 3.80E-01 3.90E-01 4.00E-01 4.10E-01 4.20E-01 4.30E-01 4.40E-01 4.50E-01 4.60E-01 4.70E-01 4.80E-01 4.90E-01 5.00E-01 5.10E-01 5.20E-01 5.30E-01 5.40E-01 5.50E-01 5.60E-01 5.70E-01 5.80E-01 5.90E-01 6.00E-01 6.10E-01 6.20E-01 6.30E-01 6.40E-01 6.50E-01 6.60E-01 6.70E-01 6.80E-01 6.90E-01 7.00E-01 7.10E-01 7.20E-01 7.30E-01 7.40E-01 7.50E-01 7.60E-01 7.70E-01 7.80E-01 7.90E-01 8.00E-01 8.10E-01 8.20E-01 8.30E-01 8.40E-01 8.50E-01 8.60E-01 8.70E-01 8.80E-01 8.90E-01 9.00E-01 9.10E-01 9.20E-01 9.30E-01 9.40E-01 9.50E-01 9.60E-01 9.70E-01 9.80E-01 9.90E-01 1.00E+00];

(1) FA5010: D=0.13mm, T=5.0s; *ϕ*=

[2.81E-04 4.16E-04 5.51E-04 6.83E-04 7.95E-04 8.87E-04 9.68E-04 1.04E-03 1.10E-03 1.16E-03 1.21E-03 1.25E-03 1.29E-03 1.33E-03 1.36E-03 1.39E-03 1.41E-03 1.44E-03 1.46E-03 1.48E-03 1.50E-03 1.51E-03 1.52E-03 1.53E-03 1.54E-03 1.53E-03 1.51E-03 1.49E-03 1.44E-03 1.39E-03 1.32E-03 1.24E-03 1.14E-03 1.03E-03 9.13E-04 7.84E-04 6.51E-04 5.14E-04 3.77E-04 2.40E-04 9.96E-05 -5.68E-05 -2.00E-04 -3.31E-04 -4.49E-04 -5.54E-04 -6.48E-04 -7.14E-04 -7.65E-04 -8.08E-04 -8.44E-04 -8.76E-04 -9.04E-04 -9.30E-04 -9.54E-04 -9.77E-04 -9.98E-04 -1.02E-03 -1.04E-03 -1.05E-03 -1.07E-03 -1.09E-03 -1.10E-03 -1.11E-03 -1.12E-03 -1.13E-03 -1.13E-03 -1.14E-03 -1.14E-03 -1.14E-03 -1.14E-03 -1.14E-03 -1.14E-03 -1.13E-03 -1.12E-03 -1.11E-03 -1.10E-03 -1.09E-03 -1.08E-03 -1.07E-03 -1.05E-03 -1.03E-03 -1.01E-03 -9.86E-04 -9.60E-04 -9.30E-04 -8.95E-04 -8.56E-04 -8.12E-04 -7.62E-04 -7.07E-04 -6.45E-04 -5.77E-04 -5.02E-04 -4.21E-04 -3.32E-04 -2.36E-04 -1.13E-04 1.47E-05 1.46E-04 2.81E-04]

(2) FA7515: D=0.13mm, T=7.5s; *ϕ*=

[2.01E-04 2.78E-04 3.53E-04 4.25E-04 4.87E-04 5.30E-04 5.68E-04 6.02E-04 6.33E-04 6.63E-04 6.92E-04 7.21E-04 7.51E-04 7.82E-04 8.15E-04 8.50E-04 8.86E-04 9.24E-04 9.61E-04 9.98E-04 1.03E-03 1.06E-03 1.09E-03 1.12E-03 1.14E-03 1.14E-03 1.14E-03 1.12E-03 1.08E-03 1.03E-03 9.69E-04 8.90E-04 8.02E-04 7.07E-04 6.07E-04 5.05E-04 4.04E-04 3.04E-04 2.08E-04 1.16E-04 2.10E-05 -7.26E-05 -1.54E-04 -2.26E-04 -2.87E-04 -3.42E-04 -3.89E-04 -4.24E-04 -4.46E-04 -4.66E-04 -4.84E-04 -5.03E-04 -5.21E-04 -5.40E-04 -5.58E-04 -5.77E-04 -5.95E-04 -6.13E-04 -6.30E-04 -6.45E-04 -6.60E-04 -6.72E-04 -6.83E-04 -6.92E-04 -6.99E-04 -7.04E-04 -7.08E-04 -7.11E-04 -7.12E-04 -7.12E-04 -7.12E-04 -7.11E-04 -7.06E-04 -7.00E-04 -6.93E-04 -6.85E-04 -6.77E-04 -6.68E-04 -6.59E-04 -6.48E-04 -6.37E-04 -6.26E-04 -6.13E-04 -5.98E-04 -5.81E-04 -5.61E-04 -5.39E-04 -5.13E-04 -4.85E-04 -4.52E-04 -4.16E-04 -3.76E-04 -3.32E-04 -2.84E-04 -2.31E-04 -1.74E-04 -1.09E-04 -3.32E-05 4.41E-05 1.22E-04 2.01E-04]

(3) CA5010: D=0.46mm, T=5.0s; *ϕ*=

[1.32E-05 1.55E-05 1.84E-05 2.23E-05 2.80E-05 3.65E-05 4.74E-05 6.18E-05 8.14E-05 1.07E-04 1.40E-04 1.81E-04 2.29E-04 2.84E-04 3.44E-04 4.09E-04 4.74E-04 5.37E-04 5.95E-04 6.44E-04 6.82E-04 7.06E-04 7.18E-04 7.14E-04 6.92E-04 6.54E-04 6.04E-04 5.43E-04 4.76E-04 4.06E-04 3.36E-04 2.69E-04 2.08E-04 1.55E-04 1.10E-04 7.40E-05 4.62E-05 2.56E-05 1.07E-05 6.10E-07 -5.29E-06 -8.61E-06 -1.06E-05 -1.20E-05 -1.37E-05 -1.59E-05 -1.90E-05 -2.32E-05 -2.89E-05 -3.60E-05 -4.39E-05 -5.23E-05 -6.18E-05 -7.19E-05 -8.23E-05 -9.28E-05 -1.03E-04 -1.12E-04 -1.21E-04 -1.29E-04 -1.35E-04 -1.40E-04 -1.44E-04 -1.47E-04 -1.49E-04 -1.50E-04 -1.51E-04 -1.51E-04 -1.50E-04 -1.49E-04 -1.49E-04 -1.48E-04 -1.46E-04 -1.44E-04 -1.42E-04 -1.41E-04 -1.39E-04 -1.37E-04 -1.35E-04 -1.33E-04 -1.29E-04 -1.26E-04 -1.21E-04 -1.16E-04 -1.10E-04 -1.02E-04 -9.43E-05 -8.53E-05 -7.56E-05 -6.54E-05 -5.50E-05 -4.45E-05 -3.44E-05 -2.49E-05 -1.56E-05 -7.56E-06 -1.06E-06 3.99E-06 7.82E-06 1.08E-05 1.32E-05]

(4) CA7515: D=0.46mm, T=7.5s *ϕ*=

[5.36E-07 5.67E-07 7.80E-07 1.36E-06 2.66E-06 5.32E-06 1.01E-05 1.75E-05 2.86E-05 4.45E-05 6.57E-05 9.26E-05 1.25E-04 1.63E-04 2.04E-04 2.47E-04 2.90E-04 3.31E-04 3.67E-04 3.95E-04 4.14E-04 4.23E-04 4.22E-04 4.12E-04 3.90E-04 3.60E-04 3.22E-04 2.80E-04 2.35E-04 1.91E-04 1.50E-04 1.12E-04 8.00E-05 5.41E-05 3.41E-05 1.98E-05 1.02E-05 4.32E-06 1.03E-06 -3.33E-07 -6.87E-07 -6.41E-07 -5.17E-07 -4.49E-07 -4.90E-07 -6.91E-07 -1.15E-06 -2.00E-06 -3.43E-06 -5.60E-06 -8.62E-06 -1.22E-05 -1.64E-05 -2.11E-05 -2.61E-05 -3.13E-05 -3.64E-05 -4.13E-05 -4.58E-05 -4.98E-05 -5.32E-05 -5.59E-05 -5.81E-05 -5.97E-05 -6.08E-05 -6.15E-05 -6.19E-05 -6.20E-05 -6.19E-05 -6.17E-05 -6.14E-05 -6.11E-05 -6.05E-05 -5.98E-05 -5.91E-05 -5.83E-05 -5.75E-05 -5.66E-05 -5.54E-05 -5.40E-05 -5.23E-05 -5.02E-05 -4.78E-05 -4.48E-05 -4.14E-05 -3.75E-05 -3.33E-05 -2.88E-05 -2.41E-05 -1.95E-05 -1.50E-05 -1.10E-05 -7.42E-06 -4.44E-06 -2.14E-06 -6.32E-07 2.10E-07 5.70E-07 6.39E-07 5.87E-07 5.36E-07]

Figure 5. *U/U_m_* is given by 2^nd^ Stokes flow of Liu and Sato (2006)

*t/T*=[0.00E+00 1.00E-02 2.00E-02 3.00E-02 4.00E-02 5.00E-02 6.00E-02 7.00E-02 8.00E-02 9.00E-02 1.00E-01 1.10E-01 1.20E-01 1.30E-01 1.40E-01 1.50E-01 1.60E-01 1.70E-01 1.80E-01 1.90E-01 2.00E-01 2.10E-01 2.20E-01 2.30E-01 2.40E-01 2.50E-01 2.60E-01 2.70E-01 2.80E-01 2.90E-01 3.00E-01 3.10E-01 3.20E-01 3.30E-01 3.40E-01 3.50E-01 3.60E-01 3.70E-01 3.80E-01 3.90E-01 4.00E-01 4.10E-01 4.20E-01 4.30E-01 4.40E-01 4.50E-01 4.60E-01 4.70E-01 4.80E-01 4.90E-01 5.00E-01 5.10E-01 5.20E-01 5.30E-01 5.40E-01 5.50E-01 5.60E-01 5.70E-01 5.80E-01 5.90E-01 6.00E-01 6.10E-01 6.20E-01 6.30E-01 6.40E-01 6.50E-01 6.60E-01 6.70E-01 6.80E-01 6.90E-01 7.00E-01 7.10E-01 7.20E-01 7.30E-01 7.40E-01 7.50E-01 7.60E-01 7.70E-01 7.80E-01 7.90E-01 8.00E-01 8.10E-01 8.20E-01 8.30E-01 8.40E-01 8.50E-01 8.60E-01 8.70E-01 8.80E-01 8.90E-01 9.00E-01 9.10E-01 9.20E-01 9.30E-01 9.40E-01 9.50E-01 9.60E-01 9.70E-01 9.80E-01 9.90E-01 1.00E+00];

(1) FA5010: D=0.13mm, T=5.0s; *ϕ/ϕ_m_*=

[1.83E-01 2.71E-01 3.58E-01 4.45E-01 5.18E-01 5.77E-01 6.30E-01 6.77E-01 7.18E-01 7.54E-01 7.87E-01 8.15E-01 8.41E-01 8.64E-01 8.84E-01 9.03E-01 9.21E-01 9.37E-01 9.51E-01 9.64E-01 9.74E-01 9.82E-01 9.91E-01 9.98E-01 1.00E+00 9.96E-01 9.85E-01 9.67E-01 9.40E-01 9.04E-01 8.60E-01 8.06E-01 7.44E-01 6.73E-01 5.94E-01 5.11E-01 4.24E-01 3.35E-01 2.45E-01 1.56E-01 6.48E-02 -3.69E-02 -1.31E-01 -2.15E-01 -2.92E-01 -3.61E-01 -4.22E-01 -4.65E-01 -4.98E-01 -5.26E-01 -5.50E-01 -5.70E-01 -5.89E-01 -6.05E-01 -6.21E-01 -6.36E-01 -6.50E-01 -6.63E-01 -6.75E-01 -6.87E-01 -6.97E-01 -7.06E-01 -7.15E-01 -7.22E-01 -7.28E-01 -7.33E-01 -7.37E-01 -7.40E-01 -7.42E-01 -7.43E-01 -7.44E-01 -7.44E-01 -7.41E-01 -7.36E-01 -7.31E-01 -7.25E-01 -7.18E-01 -7.11E-01 -7.03E-01 -6.94E-01 -6.83E-01 -6.71E-01 -6.57E-01 -6.42E-01 -6.25E-01 -6.05E-01 -5.83E-01 -5.57E-01 -5.29E-01 -4.96E-01 -4.60E-01 -4.20E-01 -3.76E-01 -3.27E-01 -2.74E-01 -2.16E-01 -1.53E-01 -7.37E-02 9.59E-03 9.53E-02 1.83E-01]

(2) FA7515: D=0.13mm, T=7.5s; *ϕ/ϕ_m_*=

[1.76E-01 2.43E-01 3.09E-01 3.72E-01 4.27E-01 4.64E-01 4.97E-01 5.27E-01 5.55E-01 5.81E-01 6.06E-01 6.32E-01 6.58E-01 6.85E-01 7.14E-01 7.44E-01 7.76E-01 8.09E-01 8.42E-01 8.74E-01 9.05E-01 9.32E-01 9.58E-01 9.80E-01 9.95E-01 1.00E+00 9.94E-01 9.77E-01 9.47E-01 9.05E-01 8.49E-01 7.80E-01 7.02E-01 6.19E-01 5.32E-01 4.43E-01 3.54E-01 2.67E-01 1.82E-01 1.01E-01 1.84E-02 -6.36E-02 -1.35E-01 -1.98E-01 -2.52E-01 -2.99E-01 -3.41E-01 -3.71E-01 -3.90E-01 -4.08E-01 -4.24E-01 -4.40E-01 -4.56E-01 -4.73E-01 -4.89E-01 -5.05E-01 -5.22E-01 -5.37E-01 -5.52E-01 -5.65E-01 -5.78E-01 -5.89E-01 -5.98E-01 -6.06E-01 -6.12E-01 -6.17E-01 -6.20E-01 -6.23E-01 -6.24E-01 -6.24E-01 -6.24E-01 -6.23E-01 -6.19E-01 -6.13E-01 -6.07E-01 -6.00E-01 -5.93E-01 -5.85E-01 -5.77E-01 -5.68E-01 -5.58E-01 -5.48E-01 -5.37E-01 -5.24E-01 -5.09E-01 -4.92E-01 -4.72E-01 -4.50E-01 -4.24E-01 -3.96E-01 -3.64E-01 -3.29E-01 -2.91E-01 -2.48E-01 -2.02E-01 -1.53E-01 -9.55E-02 -2.91E-02 3.87E-02 1.07E-01 1.76E-01]

(3) CA5010: D=0.46mm, T=5.0s; *ϕ/ϕ_m_*=

[1.84E-02 2.16E-02 2.56E-02 3.10E-02 3.90E-02 5.09E-02 6.60E-02 8.60E-02 1.13E-01 1.49E-01 1.95E-01 2.52E-01 3.19E-01 3.95E-01 4.79E-01 5.69E-01 6.59E-01 7.47E-01 8.28E-01 8.97E-01 9.50E-01 9.83E-01 9.99E-01 9.94E-01 9.63E-01 9.11E-01 8.40E-01 7.56E-01 6.63E-01 5.65E-01 4.68E-01 3.75E-01 2.90E-01 2.16E-01 1.53E-01 1.03E-01 6.42E-02 3.57E-02 1.48E-02 8.49E-04 -7.36E-03 -1.20E-02 -1.47E-02 -1.68E-02 -1.90E-02 -2.21E-02 -2.64E-02 -3.23E-02 -4.02E-02 -5.01E-02 -6.10E-02 -7.28E-02 -8.60E-02 -1.00E-01 -1.15E-01 -1.29E-01 -1.43E-01 -1.57E-01 -1.69E-01 -1.79E-01 -1.88E-01 -1.95E-01 -2.01E-01 -2.05E-01 -2.08E-01 -2.09E-01 -2.10E-01 -2.10E-01 -2.09E-01 -2.08E-01 -2.07E-01 -2.05E-01 -2.03E-01 -2.01E-01 -1.98E-01 -1.96E-01 -1.94E-01 -1.91E-01 -1.88E-01 -1.85E-01 -1.80E-01 -1.75E-01 -1.69E-01 -1.61E-01 -1.53E-01 -1.43E-01 -1.31E-01 -1.19E-01 -1.05E-01 -9.11E-02 -7.65E-02 -6.20E-02 -4.79E-02 -3.47E-02 -2.17E-02 -1.05E-02 -1.48E-03 5.56E-03 1.09E-02 1.50E-02 1.84E-02]

(4) CA7515: D=0.46mm, T=7.5s *ϕ/ϕ_m_*=

[1.26E-03 1.34E-03 1.84E-03 3.20E-03 6.28E-03 1.26E-02 2.38E-02 4.12E-02 6.76E-02 1.05E-01 1.55E-01 2.19E-01 2.95E-01 3.84E-01 4.81E-01 5.83E-01 6.85E-01 7.81E-01 8.65E-01 9.32E-01 9.78E-01 9.99E-01 9.97E-01 9.71E-01 9.21E-01 8.49E-01 7.60E-01 6.61E-01 5.56E-01 4.51E-01 3.53E-01 2.64E-01 1.89E-01 1.28E-01 8.06E-02 4.67E-02 2.41E-02 1.02E-02 2.42E-03 -7.86E-04 -1.62E-03 -1.51E-03 -1.22E-03 -1.06E-03 -1.16E-03 -1.63E-03 -2.71E-03 -4.73E-03 -8.11E-03 -1.32E-02 -2.03E-02 -2.88E-02 -3.86E-02 -4.97E-02 -6.16E-02 -7.39E-02 -8.60E-02 -9.75E-02 -1.08E-01 -1.17E-01 -1.25E-01 -1.32E-01 -1.37E-01 -1.41E-01 -1.44E-01 -1.45E-01 -1.46E-01 -1.46E-01 -1.46E-01 -1.46E-01 -1.45E-01 -1.44E-01 -1.43E-01 -1.41E-01 -1.40E-01 -1.38E-01 -1.36E-01 -1.33E-01 -1.31E-01 -1.27E-01 -1.23E-01 -1.19E-01 -1.13E-01 -1.06E-01 -9.77E-02 -8.86E-02 -7.86E-02 -6.80E-02 -5.70E-02 -4.60E-02 -3.55E-02 -2.59E-02 -1.75E-02 -1.05E-02 -5.04E-03 -1.49E-03 4.96E-04 1.34E-03 1.51E-03 1.39E-03 1.26E-03]

Figure 6. ‘*q_n_*(Exp.)’ is given by O’Donoghue and Wright (2004b); ‘*q_L_*’ is given by Liu and Sato (2006);

*q_on_*(*y*)=$\int_{0}^{Tc} S(y,t)U_{B}(y,t)dt/T$, *q_off_*(*y*)=$\int_{Tc}^{T} S(y,t)U_{B}(y,t)dt/T$ and *q_n_*=*q_on_*+*q_off_*; *S* is given by Equation (5) and *U_B_* is given by Equation (4)

*y*=[-1.00E-02 -9.50E-03 -9.00E-03 -8.50E-03 -8.00E-03 -7.50E-03 -7.00E-03 -6.50E-03 -6.00E-03 -5.50E-03 -5.00E-03 -4.50E-03 -4.00E-03 -3.50E-03 -3.00E-03 -2.50E-03 -2.00E-03 -1.50E-03 -1.00E-03 -5.00E-04 0.00E+00 5.00E-04 1.00E-03 1.50E-03 2.00E-03 2.50E-03 3.00E-03 3.50E-03 4.00E-03 4.50E-03 5.00E-03 5.50E-03 6.00E-03 6.50E-03 7.00E-03 7.50E-03 8.00E-03 8.50E-03 9.00E-03 9.50E-03 1.00E-02 1.10E-02 1.20E-02 1.30E-02 1.40E-02 1.50E-02 1.60E-02 1.70E-02 1.80E-02 1.90E-02 2.00E-02 2.10E-02 2.20E-02 2.30E-02 2.40E-02 2.50E-02 2.60E-02 2.70E-02 2.80E-02 2.90E-02 3.00E-02]

Figure 7. ‘Exp’ is given by Ribberink and Chen (1993), Hassan and Ribberink (2005) and Ahmed and Sato (2003); ‘Pres.’ is given by Equation (7); ‘R98’ is given by Ribberink (1998); ‘N06’ is given by Nielsen (2006); ‘W04’ is given by Watanabe and Sato (2004); ‘S06’ is given by Silva et al (2006).

*U_m_*=[1.00E-01 2.00E-01 3.00E-01 4.00E-01 5.00E-01 6.00E-01 7.00E-01 8.00E-01 9.00E-01 1.00E+00 1.05E+00 1.10E+00 1.15E+00 1.20E+00 1.25E+00 1.30E+00 1.35E+00 1.40E+00 1.45E+00 1.50E+00 1.55E+00 1.60E+00 1.65E+00 1.70E+00 1.75E+00 1.80E+00]

Figure 8.

(1) Present model; it is given by Equation (7)

(2) Watanabe and Sato (2004); it is given by Watanabe and Sato (2004)

(3) Silva et al. (2006); it is given by Silva et al. (2006)

*U_m_*=[1.00E-01 2.00E-01 3.00E-01 4.00E-01 5.00E-01 6.00E-01 7.00E-01 8.00E-01 9.00E-01 1.00E+00 1.05E+00 1.10E+00 1.15E+00 1.20E+00 1.25E+00 1.30E+00 1.35E+00 1.40E+00 1.45E+00 1.50E+00 1.55E+00 1.60E+00 1.65E+00 1.70E+00 1.75E+00 1.80E+00]

Figure 9. ‘Pres.’ is given by Equation (7); ‘R98’ is given by Ribberink (1998); ‘N06’ is given by Nielsen (2006).

*t/T*=[0.00E+00 1.00E-02 2.00E-02 3.00E-02 4.00E-02 5.00E-02 6.00E-02 7.00E-02 8.00E-02 9.00E-02 1.00E-01 1.10E-01 1.20E-01 1.30E-01 1.40E-01 1.50E-01 1.60E-01 1.70E-01 1.80E-01 1.90E-01 2.00E-01 2.10E-01 2.20E-01 2.30E-01 2.40E-01 2.50E-01 2.60E-01 2.70E-01 2.80E-01 2.90E-01 3.00E-01 3.10E-01 3.20E-01 3.30E-01 3.40E-01 3.50E-01 3.60E-01 3.70E-01 3.80E-01 3.90E-01 4.00E-01 4.10E-01 4.20E-01 4.30E-01 4.40E-01 4.50E-01 4.60E-01 4.70E-01 4.80E-01 4.90E-01 5.00E-01 5.10E-01 5.20E-01 5.30E-01 5.40E-01 5.50E-01 5.60E-01 5.70E-01 5.80E-01 5.90E-01 6.00E-01 6.10E-01 6.20E-01 6.30E-01 6.40E-01 6.50E-01 6.60E-01 6.70E-01 6.80E-01 6.90E-01 7.00E-01 7.10E-01 7.20E-01 7.30E-01 7.40E-01 7.50E-01 7.60E-01 7.70E-01 7.80E-01 7.90E-01 8.00E-01 8.10E-01 8.20E-01 8.30E-01 8.40E-01 8.50E-01 8.60E-01 8.70E-01 8.80E-01 8.90E-01 9.00E-01 9.10E-01 9.20E-01 9.30E-01 9.40E-01 9.50E-01 9.60E-01 9.70E-01 9.80E-01 9.90E-01 1.00E+00];

Figure 10. *ϕ/ϕ_m_* is given by Equation (7); *U/U_m_* is given by Sawtooth flow of van der et al A (2010a)

*t/T*=[0.00E+00 1.00E-02 2.00E-02 3.00E-02 4.00E-02 5.00E-02 6.00E-02 7.00E-02 8.00E-02 9.00E-02 1.00E-01 1.10E-01 1.20E-01 1.30E-01 1.40E-01 1.50E-01 1.60E-01 1.70E-01 1.80E-01 1.90E-01 2.00E-01 2.10E-01 2.20E-01 2.30E-01 2.40E-01 2.50E-01 2.60E-01 2.70E-01 2.80E-01 2.90E-01 3.00E-01 3.10E-01 3.20E-01 3.30E-01 3.40E-01 3.50E-01 3.60E-01 3.70E-01 3.80E-01 3.90E-01 4.00E-01 4.10E-01 4.20E-01 4.30E-01 4.40E-01 4.50E-01 4.60E-01 4.70E-01 4.80E-01 4.90E-01 5.00E-01 5.10E-01 5.20E-01 5.30E-01 5.40E-01 5.50E-01 5.60E-01 5.70E-01 5.80E-01 5.90E-01 6.00E-01 6.10E-01 6.20E-01 6.30E-01 6.40E-01 6.50E-01 6.60E-01 6.70E-01 6.80E-01 6.90E-01 7.00E-01 7.10E-01 7.20E-01 7.30E-01 7.40E-01 7.50E-01 7.60E-01 7.70E-01 7.80E-01 7.90E-01 8.00E-01 8.10E-01 8.20E-01 8.30E-01 8.40E-01 8.50E-01 8.60E-01 8.70E-01 8.80E-01 8.90E-01 9.00E-01 9.10E-01 9.20E-01 9.30E-01 9.40E-01 9.50E-01 9.60E-01 9.70E-01 9.80E-01 9.90E-01 1.00E+00];

Figure 11. *q_on_*(*y*)=$\int_{0}^{Tc} S(y,t)U_{B}(y,t)dt/T$, *q_off_*(*y*)=$\int_{Tc}^{T} S(y,t)U_{B}(y,t)dt/T$ and *q_n_*=*q_on_*+*q_off_*; *S* is given by Equation (5) and *U_B_* is given by Equation (4)

*y*=[-1.00E-02 -9.50E-03 -9.00E-03 -8.50E-03 -8.00E-03 -7.50E-03 -7.00E-03 -6.50E-03 -6.00E-03 -5.50E-03 -5.00E-03 -4.50E-03 -4.00E-03 -3.50E-03 -3.00E-03 -2.50E-03 -2.00E-03 -1.50E-03 -1.00E-03 -5.00E-04 0.00E+00 5.00E-04 1.00E-03 1.50E-03 2.00E-03 2.50E-03 3.00E-03 3.50E-03 4.00E-03 4.50E-03 5.00E-03 5.50E-03 6.00E-03 6.50E-03 7.00E-03 7.50E-03 8.00E-03 8.50E-03 9.00E-03 9.50E-03 1.00E-02 1.10E-02 1.20E-02 1.30E-02 1.40E-02 1.50E-02 1.60E-02 1.70E-02 1.80E-02 1.90E-02 2.00E-02 2.10E-02 2.20E-02 2.30E-02 2.40E-02 2.50E-02 2.60E-02 2.70E-02 2.80E-02 2.90E-02 3.00E-02]

Figure 12. ‘Exp’ is given by Watanabe and Sato (2004) and vander A et al (2010a); ‘Pres.’ is given by Equation (7); ‘W04’ is given by Watanabe and Sato (2004); ‘S06’ is given by Silva et al (2006).

*U_m_*=[1.00E-01 2.00E-01 3.00E-01 4.00E-01 5.00E-01 6.00E-01 7.00E-01 8.00E-01 9.00E-01 1.00E+00 1.05E+00 1.10E+00 1.15E+00 1.20E+00 1.25E+00 1.30E+00 1.35E+00 1.40E+00 1.45E+00 1.50E+00 1.55E+00 1.60E+00 1.65E+00 1.70E+00 1.75E+00 1.80E+00]

Figure 13.

(1) Present model; it is given by Equation (7)

(2) Watanabe and Sato (2004); it is given by Watanabe and Sato (2004)

(3) Silva et al. (2006); it is given by Silva et al. (2006)

*U_m_*=[1.00E-01 2.00E-01 3.00E-01 4.00E-01 5.00E-01 6.00E-01 7.00E-01 8.00E-01 9.00E-01 1.00E+00 1.05E+00 1.10E+00 1.15E+00 1.20E+00 1.25E+00 1.30E+00 1.35E+00 1.40E+00 1.45E+00 1.50E+00 1.55E+00 1.60E+00 1.65E+00 1.70E+00 1.75E+00 1.80E+00]

Figure 14. ‘Pres.’ is given by Equation (7); ‘R98’ is given by Ribberink (1998); ‘N06’ is given by Nielsen (2006).

*t/T*=[0.00E+00 1.00E-02 2.00E-02 3.00E-02 4.00E-02 5.00E-02 6.00E-02 7.00E-02 8.00E-02 9.00E-02 1.00E-01 1.10E-01 1.20E-01 1.30E-01 1.40E-01 1.50E-01 1.60E-01 1.70E-01 1.80E-01 1.90E-01 2.00E-01 2.10E-01 2.20E-01 2.30E-01 2.40E-01 2.50E-01 2.60E-01 2.70E-01 2.80E-01 2.90E-01 3.00E-01 3.10E-01 3.20E-01 3.30E-01 3.40E-01 3.50E-01 3.60E-01 3.70E-01 3.80E-01 3.90E-01 4.00E-01 4.10E-01 4.20E-01 4.30E-01 4.40E-01 4.50E-01 4.60E-01 4.70E-01 4.80E-01 4.90E-01 5.00E-01 5.10E-01 5.20E-01 5.30E-01 5.40E-01 5.50E-01 5.60E-01 5.70E-01 5.80E-01 5.90E-01 6.00E-01 6.10E-01 6.20E-01 6.30E-01 6.40E-01 6.50E-01 6.60E-01 6.70E-01 6.80E-01 6.90E-01 7.00E-01 7.10E-01 7.20E-01 7.30E-01 7.40E-01 7.50E-01 7.60E-01 7.70E-01 7.80E-01 7.90E-01 8.00E-01 8.10E-01 8.20E-01 8.30E-01 8.40E-01 8.50E-01 8.60E-01 8.70E-01 8.80E-01 8.90E-01 9.00E-01 9.10E-01 9.20E-01 9.30E-01 9.40E-01 9.50E-01 9.60E-01 9.70E-01 9.80E-01 9.90E-01 1.00E+00];

Figure 15. *ϕ/ϕ_m_* is given by Equation (7); *U/U_m_* is given by mixed flow of Abreu et al (2010)

*t/T*=[0.00E+00 1.00E-02 2.00E-02 3.00E-02 4.00E-02 5.00E-02 6.00E-02 7.00E-02 8.00E-02 9.00E-02 1.00E-01 1.10E-01 1.20E-01 1.30E-01 1.40E-01 1.50E-01 1.60E-01 1.70E-01 1.80E-01 1.90E-01 2.00E-01 2.10E-01 2.20E-01 2.30E-01 2.40E-01 2.50E-01 2.60E-01 2.70E-01 2.80E-01 2.90E-01 3.00E-01 3.10E-01 3.20E-01 3.30E-01 3.40E-01 3.50E-01 3.60E-01 3.70E-01 3.80E-01 3.90E-01 4.00E-01 4.10E-01 4.20E-01 4.30E-01 4.40E-01 4.50E-01 4.60E-01 4.70E-01 4.80E-01 4.90E-01 5.00E-01 5.10E-01 5.20E-01 5.30E-01 5.40E-01 5.50E-01 5.60E-01 5.70E-01 5.80E-01 5.90E-01 6.00E-01 6.10E-01 6.20E-01 6.30E-01 6.40E-01 6.50E-01 6.60E-01 6.70E-01 6.80E-01 6.90E-01 7.00E-01 7.10E-01 7.20E-01 7.30E-01 7.40E-01 7.50E-01 7.60E-01 7.70E-01 7.80E-01 7.90E-01 8.00E-01 8.10E-01 8.20E-01 8.30E-01 8.40E-01 8.50E-01 8.60E-01 8.70E-01 8.80E-01 8.90E-01 9.00E-01 9.10E-01 9.20E-01 9.30E-01 9.40E-01 9.50E-01 9.60E-01 9.70E-01 9.80E-01 9.90E-01 1.00E+00];

Figure 16. ‘Exp.’ is given by Dong *et al* (2013); ‘Pres.’ is given by Equation (7); ‘R98’ is given by Ribberink (1998); ‘W04’ is given by Watanabe and Sato (2004); ‘S06’ is given by Silva et al (2006); ‘N06’ is given by Nielsen (2006).

*U_m_*=[1.00E-01 2.00E-01 3.00E-01 4.00E-01 5.00E-01 6.00E-01 7.00E-01 8.00E-01 9.00E-01 1.00E+00 1.05E+00 1.10E+00 1.15E+00 1.20E+00 1.25E+00 1.30E+00 1.35E+00 1.40E+00 1.45E+00 1.50E+00 1.55E+00 1.60E+00 1.65E+00 1.70E+00 1.75E+00 1.80E+00]

Figure 17. ‘Exp.’ is given by Dong *et al* (2013); ‘Pres.’ is given by Equation (7)

*U_m_*=[1.00E-01 2.00E-01 3.00E-01 4.00E-01 5.00E-01 6.00E-01 7.00E-01 8.00E-01 9.00E-01 1.00E+00 1.05E+00 1.10E+00 1.15E+00 1.20E+00 1.25E+00 1.30E+00 1.35E+00 1.40E+00 1.45E+00 1.50E+00 1.55E+00 1.60E+00 1.65E+00 1.70E+00 1.75E+00 1.80E+00]

Figure 18. Δ is given by Equation (14)

*t/T*=[0.00E+00 1.00E-02 2.00E-02 3.00E-02 4.00E-02 5.00E-02 6.00E-02 7.00E-02 8.00E-02 9.00E-02 1.00E-01 1.10E-01 1.20E-01 1.30E-01 1.40E-01 1.50E-01 1.60E-01 1.70E-01 1.80E-01 1.90E-01 2.00E-01 2.10E-01 2.20E-01 2.30E-01 2.40E-01 2.50E-01 2.60E-01 2.70E-01 2.80E-01 2.90E-01 3.00E-01 3.10E-01 3.20E-01 3.30E-01 3.40E-01 3.50E-01 3.60E-01 3.70E-01 3.80E-01 3.90E-01 4.00E-01 4.10E-01 4.20E-01 4.30E-01 4.40E-01 4.50E-01 4.60E-01 4.70E-01 4.80E-01 4.90E-01 5.00E-01 5.10E-01 5.20E-01 5.30E-01 5.40E-01 5.50E-01 5.60E-01 5.70E-01 5.80E-01 5.90E-01 6.00E-01 6.10E-01 6.20E-01 6.30E-01 6.40E-01 6.50E-01 6.60E-01 6.70E-01 6.80E-01 6.90E-01 7.00E-01 7.10E-01 7.20E-01 7.30E-01 7.40E-01 7.50E-01 7.60E-01 7.70E-01 7.80E-01 7.90E-01 8.00E-01 8.10E-01 8.20E-01 8.30E-01 8.40E-01 8.50E-01 8.60E-01 8.70E-01 8.80E-01 8.90E-01 9.00E-01 9.10E-01 9.20E-01 9.30E-01 9.40E-01 9.50E-01 9.60E-01 9.70E-01 9.80E-01 9.90E-01 1.00E+00];

Figure 19. Data are collected in Table 1.

(1) View of Cartesian coordinates, van der A et al (2013); it is given by van der A et al (2013)

(2) View of log-log coordinates, van der A et al (2013); it is given by van der A et al (2013)

(3) View of Cartesian coordinates, Equation (7); it is given by Equation (7)

(4) View of log-log coordinates, Equation (7); it is given by Equation (7)
